# DNA Methylation of the t-PA Gene Differs Between Various Immune Cell Subtypes Isolated From Depressed Patients Receiving Electroconvulsive Therapy

**DOI:** 10.3389/fpsyt.2020.00571

**Published:** 2020-06-19

**Authors:** Nicole Moschny, Kirsten Jahn, Malek Bajbouj, Hannah Benedictine Maier, Matthias Ballmaier, Abdul Qayyum Khan, Christoph Pollak, Stefan Bleich, Helge Frieling, Alexandra Neyazi

**Affiliations:** ^1^Laboratory for Molecular Neurosciences, Department of Psychiatry, Social Psychiatry and Psychotherapy, Hannover Medical School, Hannover, Germany; ^2^Center for Systems Neuroscience, Hannover Graduate School for Veterinary Pathobiology, Neuroinfectiology, and Translational Medicine (HGNI), Hannover, Germany; ^3^Department of Psychiatry and Psychotherapy, Charité, Berlin, Germany; ^4^Department of Psychiatry, Social Psychiatry and Psychotherapy, Hannover Medical School, Hannover, Germany; ^5^Cell Sorting Core Facility, Hannover Medical School, Hannover, Germany

**Keywords:** brain-derived neurotrophic factor, tissue-type plasminogen activator, depression, immunology, DNA methylation, electroconvulsive therapy remission

## Abstract

**Background:**

Major depressive disorder (MDD) represents a tremendous health threat to the world’s population. Electroconvulsive therapy (ECT) is the most effective treatment option for refractory MDD patients. Ample evidence suggests brain-derived neurotrophic factor (BDNF) to play a crucial role in ECT’s mode of action. Tissue-type plasminogen activator (t-PA) and plasminogen activator inhibitor-1 (PAI-1) are involved in BDNF production.

**Hypothesis:**

The DNA methylation of gene regions encoding for t-PA and PAI-1 might be a suitable biomarker for ECT response prediction.

**Methods:**

We withdrew blood from two cohorts of treatment-resistant MDD patients receiving ECT. In the first cohort (n = 59), blood was collected at baseline only. To evaluate DNA methylation changes throughout the treatment course, we acquired a second group (n = 28) and took blood samples at multiple time points. DNA isolated from whole blood and defined immune cell subtypes (B cells, monocytes, natural killer cells, and T cells) served for epigenetic analyses.

**Results:**

Mixed linear models (corrected for multiple testing by Sidak’s post-hoc test) revealed (1) no detectable baseline blood DNA methylation differences between ECT remitters (n = 33) and non-remitters (n = 53) in the regions analyzed, but (2) a significant difference in t-PA’s DNA methylation between the investigated immune cell subtypes instead (p < 0.00001). This difference remained stable throughout the treatment course, showed no acute changes after ECT, and was independent of clinical remission.

**Conclusion:**

DNA methylation of both proteins seems to play a minor role in ECT’s mechanisms. Generally, we recommend using defined immune cell subtypes (instead of whole blood only) for DNA methylation analyses.

## Introduction

According to the World Health Organization, major depressive disorder (MDD) can, nowadays, primarily be accounted for approximately 800,000 suicide deaths per year. With >322 million individuals affected, it belongs to the most prevalent mental disorders worldwide (1). Unfortunately, the urge for appropriate therapy is being challenged by the incomplete knowledge of its etiology and pathomechanism.

“Genes load the gun and environment pulls the trigger”—an urban metaphor evolving in the last two decades—emphasizes on the interplay of nature and nurture for disease development: susceptibility to certain disorders depends on the genetic constitution, but additional environmental factors (e.g., stress, abuse or neglect) are often necessary to trigger disease onset (2–4). The latter effect is mediated by epigenetic mechanisms, including DNA/RNA methylation changes or histone modifications, that are capable of changing protein expression (4–8). Regarding MDD, no robust risk genes have been identified so far (9, 10), but various experiments underline the influence of maternal care on DNA methylation, stress reactivity, and anxiety-related behavior in mice (11). In line with these findings, Fuchikami et al. were able to distinguish healthy subjects from depressed patients by DNA methylation analysis of gene regions encoding for BDNF (brain-derived neurotrophic factor) (12)—a protein playing a pivotal role in various neuropsychiatric diseases, including depression (13–15).

Despite the huge variety of antidepressants that are available on the market, 30% to 50% of MDD patients fail to achieve complete remission (16, 17). Electroconvulsive therapy (ECT) has been stated to be the most powerful alternative in the acute treatment of refractory depression (18). A mounting body of evidence suggests ECT to modulate the immune system (19–21), and to boost peripheral neurotrophin expression (22–24). Accordingly, lowered BDNF serum levels of depressed patients (15) were restored after ECT (25). In this context, Sartorius and his group proposed blood-borne BDNF to contribute to parenchymal BDNF (26), and further animal experiments showed peripherally administered neurotrophins to promote neurogenesis and neuroplasticity in the brain (27), probably by mediating direct neuronal support (28) or modulating microglial functions (29). Impaired neuroplasticity [e.g., decreased hippocampal volumes (30), diminished dendritic ramification, or reduced numbers of neurons and oligodendrocytes (31, 32)] are a hallmark of MDD patients or at least of a subgroup thereof. ECT’s proposed pro-neurogenic properties are, therefore, considered to be of therapeutic relevance (25, 33, 34).

The enzyme t-PA (tissue-type plasminogen activator) and its inhibitor PAI-1 (plasminogen activator inhibitor-1) are implicated in the cleavage of extracellular pro-BDNF and are thus important for BDNF production (35, 36). Both proteins have additional roles in the regulation of the immune system, as in macrophage migration (37) and NF-κB activation (38). Interestingly, depressed patients show declined t-PA serum levels (39) and raised PAI-1 activity (40) when compared to healthy controls. Further evidence for their involvement in MDD arises from animal experiments that found low t-PA measures in the CNS (correlating with high levels of PAI-1) to be associated with anxiety- and depressive-like behavior (41). Additionally, pro-BDNF and t-PA concentrations were upregulated in the rat hippocampus—followed by an increase of mature BDNF—after treating rodents with electroconvulsive shocks, the animal model of ECT; intriguingly, this effect was not seen upon imipramine treatment (42). Although both proteins seem to be a link between multiple factors that are highly relevant for MDD (43, 44), their epigenetics have not been investigated in this context yet.

We are the first ones to analyze the DNA methylation of gene regions encoding for t-PA (*PLAT*) and PAI-1 (*SERPINE1*) in depressed patients undergoing a course of ECT. Importantly, previous studies in the biomarker field of psychiatric epigenetics analyzed—if merely blood was taken—the DNA methylation of the diverse mixture of immune cells only. Instead of investigating different immune cell subtypes separately, these studies tend to draw conclusions that highly depend on immune cell counts and their changes. To address this problem, we isolate peripheral blood mononuclear cells (PBMCs) from our patients and sort defined immune cell subpopulations (i.e., B cells, monocytes, natural killer (NK) cells, and T cells) using flow cytometry. Analyzing the DNA methylation of these subsets separately enables us to unravel whether there is a particular immune cell subtype that takes the lead regarding clinical outcome, thereby allowing us to get a deeper insight into ECT’s therapeutic effects. By these means, we pursue (1) to characterize ECT’s general influence on DNA methylation of defined target regions reported to be implicated in MDD, (2) to illuminate the relevance of DNA methylation at baseline or ECT-associated epigenetic changes for clinical remission, and (3) to explore the inaccuracy of DNA methylation measurements obtained from whole blood only. We hypothesize ECT to cause changes in the epigenetics of defined immune cell subtypes in target regions encoding for t-PA and PAI-1, and this effect to be of therapeutic relevance. We further suggest the DNA methylation of these regions to serve as a possible biomarker for ECT response prediction.

## Material and Methods

### Study Design

Our prospective study included two different cohorts of refractory MDD patients: the 1^st^ cohort (also referred to as cross-sectional cohort in the following; n = 67) was acquired at the Department of Psychiatry and Psychotherapy of the Charité Berlin (Germany), the 2^nd^ one (=longitudinal cohort; n = 30) at the Department of Psychiatry, Social Psychiatry and Psychotherapy of the Hannover Medical School (Germany). The study was approved by the Ethics Committee of the Charité (EK-224-05c) and the Hannover Medical School (2842-2015) and followed the ethical principles of the Declaration of Helsinki (1964), including its later amendments. All participants gave written informed consent before study inclusion.

### Patients

MDD was diagnosed according to the International Statistical Classification of Diseases and Related Health Problems 10^th^ Revision (ICD-10) and disease severity assessed before and after the 1^st^, 4^th^, the last, and the maintenance ECTs by using either the Hamilton Rating Scale for Depression (HAM-D), or the Montgomery-Åsberg Depression Rating Scale (MADRS) and the Beck’s Depression Inventory (BDI-II). The Mini-Mental State Examination (MMSE) was conducted at the same time points. A decline in HAM-D or MADRS scores of ≥50% was interpreted as response, and values of ≤7 (HAM-D) or ≤10 (MADRS) as remission. Non-responsiveness to two state-of-the-art antidepressants (after two weeks of treatment with adequate dosages, respectively) was classified as treatment-resistance. Patients diseased with an infectious, autoimmune, or schizophrenic disorder were excluded. Further exclusion criteria were heightened levels of CRP (C-reactive protein) or pronounced leukocytosis.

### ECT Application and Sample Collection

As it was standard clinical practice, ECT was applied three times weekly for up to four weeks. Ultra-brief pulse devices [Mecta 5000Q and Thymatron^®^ System IV (Somatic LLC)] served for right unilateral electrical stimulation. Seizure threshold was assessed upon the first ECT session (age-based method), and the stimulus intensity increased if the seizure was insufficient (i.e., showing an EEG seizure activity below 30 sec or a motoric response shorter than 20 sec). Bilateral ECT was considered if the patient did not show any improvement after two consecutive weeks of treatment. Anesthesia was obtained with propofol or etomidate (cross-sectional cohort) or with methohexital and remifentanil (longitudinal cohort). Succinylcholine served for muscle relaxation in all study participants. Fasting blood samples were withdrawn (1) in the cross-sectional cohort: directly before starting the therapy and (2) in the longitudinal group (which served for the evaluation of DNA methylation changes throughout the full time course of ECT): before (8–10 a.m.) and 15 min after the 1^st^, the 4^th^, and the last ECT. Four follow up time points (before and after the 1^st^ and 4^th^ maintenance ECT) were included. Blood samples were collected in 2K EDTA-Gel S-Monovettes^®^ (SARSTEDT AG & Co. KG) and stored at 4°C until further processing (maximum of 3 h).

### Sample Processing

#### Blood

The blood from the cross-sectional cohort was directly used for DNA isolation. EDTA-blood from the longitudinal cohort served for PBMC isolation by density centrifugation as described elsewhere (45). Following the recommendations of Mallone et al. (46), some changes have been applied to the latter protocol; a complete description of the procedure is provided in the **Supplementary Material**.

#### PBMCs - Thawing, Staining, and Flow Cytometry

Frozen PBMCs were thawed based on a protocol reported elsewhere (47). Changes have been made to the latter procedure; a detailed explanation is given in the **Supplements**. After thawing, cells were stained as instructed by BioLegend’s staining protocol^1^ using 10% human serum in FACS buffer [=1% BSA (SIGMA-ALDRICH Co.) and 1 mM UltraPure™ EDTA (Invitrogen AG) in PBS (w/o Ca^2+^ and Mg^2+^; Biochrom GmbH)] as a blocking solution, 7-AAD (1:100, BioLegend) as a viability marker and six different antibodies for the identification of defined immune cell subtypes. Immune cell populations of interest were subsequently sorted using a BD FACSAria™ Fusion flow cytometer and the BD FACSDiva™ 8.0.1. Software (Becton, Dickinson Biosciences). Sorted cells were centrifuged (900*g*, 5 min, 4°C), the supernatant discarded and kept in 500 µl RNAprotect Cell Reagent (QIAGEN N.V.) until further processing (up to one week). Importantly, cells were kept at 4°C and treated under sterile conditions throughout the whole process (i.e., staining, sorting, storing). Further information regarding the procedure is provided in the **Supplementary Material** (**Table S1**). The sorting layout is depicted in **Figure S1**.

### DNA Isolation

#### Genomic DNA Isolation From Whole Blood

For genomic DNA (gDNA) isolation, aliquoted blood samples were thawed at 4°C overnight and 200 µl of each sample incubated with 15 µl dissolved Proteinase K (NucleoMag^®^ Blood 200 µl Kit, Macherey-Nagel) for 1 h (RT). The following procedure was performed as instructed by the NucleoMag^®^ Blood 200 µl Kit manual using an automated program designed for the Biomek^®^ NXp pipet robot (Beckman Coulter GmbH). Isolated gDNA was stored at −80°C until further processing or directly used for bisulfite conversion.

#### Genomic DNA Isolation From PBMCs

gDNA of PBMCs and sorted immune cell subtypes was isolated using the AllPrep DNA/RNA 96 Kit (QIAGEN N.V.). Minor changes have been made to the recommended procedure; a detailed description is to be found in the **Supplements**.

### Bisulfite Sequencing

#### Bisulfite Conversion

Bisulfite conversion of gDNA—extracted from blood, PBMCs, and sorted cells—was performed following the protocol of the EpiTect 96 Bisulfite Kit (QIAGEN N.V.). Converted DNA was stored at −20°C until further processing or directly used for DNA amplification.

#### DNA Amplification

Primers for fragments of interest (**Supplementary Material, Table S2**) were established using different types of software [Methyl Primer Express V1.0 (Applied Biosystems™), Geneious Pro 5.6.7. (Biomatters Ltd)] and online tools {Ensembl^2^, SNPCheck V3^3^, NetPrimer^4^, Metabion Biocalculator^5^, Match 1.0 [BIOBASE GmbH (48)]^6^}. Several research reports (49–61) were used as a rationale to define the genomic features we were primarily interested in. Correct fragment size was confirmed by gel electrophoresis using a 1% agarose gel (PEQLAB Biotechnologie GmbH). To amplify patient samples, 1 to 3 µl of bisulfite-converted DNA (of a sample each) was mixed with 5 µl HotStarTaq^®^ Master Mix, 1.2 to 3.2 µl RNase free water (both QIAGEN N.V.) and 0.4 µl of the forward and reverse primer (Metabion GmbH), respectively. For amplification, the established PCR programs (see **Supplementary Material, Table S3**) were used, and the resulting products kept at 4°C to 12°C until clean up. Automated clean up (Biomek^®^ NXp pipet robot) of the amplified fragments followed the instructions of the Agencourt AMPureXP Kit (Beckman Coulter GmbH) using CleanPCR magnetic beads (Clean NA). Eluted DNA was kept at 4°C until sequencing (up to two weeks).

#### Sanger Sequencing

DNA concentration of the amplified samples was measured with DeNovix DS-11 FX+ (DeNovix Inc.). A maximum of 30 ng bisulfite-converted DNA (per sample) was mixed with 2 µl Big Dye^®^ Sequencing Buffer, 0.5 µl Big Dye^®^ Terminator v.3.1 (both Applied Biosystems™), 0.6 µl forward or reverse primer of the respective fragment and a various amount (6.9 µl minus volume of DNA sample needed) of RNase free water per sample each. After PCR (see **Table S4**), fragments were automatically washed using the Biomek^®^ NXp pipet robot and CleanDTR magnetic beads (CleanNA) following the protocol of the Agencourt^®^ CleanSEQ^®^ Kit (Beckman Coulter GmbH). 15 µl of HI-DI™ Formamide (Applied Biosystems™) was added to each sample, and the sequences analyzed by using a HITACHI 3500xL Genetic Analyzer and the 3500 Series Data Collection Software 2 (both Applied Biosystems™). The quality of the obtained sequences was assessed using the Sequence Scanner 2 Software (Applied Biosystems™). Only sequences above the threshold of >20 (QV) and a continuous read length close to the expected fragment size were included in the statistical analysis. All t-PA fragments that are listed in **Table S2** were sequenced in the cross-sectional cohort, but—to analyze the DNA methylation changes throughout the treatment course—only fragments showing significant results were repeated in the longitudinal cohort (fragment t-PA 2,4,5). In this context, blood-DNA of all time points was sequenced, whereas DNA isolated from immune cells was used from the 1^st^ and last ECT only. Although PAI-1’s DNA methylation was showing a very low inter-individual variance, one fragment (PAI-1 2), which serves as a binding site for multiple transcription factors, was repeatedly sequenced in the blood samples of the longitudinal cohort to obtain a larger group size for subsequent DNA methylation analyses.

### Data Processing

The Epigenetic Sequencing Methylation Analysis Software [ESME (62), Epigenomics AG] served for DNA methylation analyses. Statistical analyses were performed with IBM SPSS Statistics 25 (IBM Germany GmbH), and data presented with SPSS and GraphPad Prism 8 (Graph Pad Inc.).

#### Statistics

Patients’ demographics were normally distributed. Fisher’s exact tests and T tests were used for the analysis of clinical baseline differences between ECT remitters and non-remitters. Bisulfite sequencing yielded methylation values of 39 single CpG sites within the t-PA gene and 35 CpGs within the PAI-1 gene. Before analysis, initial quality checks for the exclusion of potentially unreliable measurements were performed: CpGs with an inter-individual variance below 1% were excluded, leaving 35 CpG sites for the analysis of t-PA. DNA methylation variance of almost all CpGs (32 of 35) within the sequenced PAI-1 gene regions was lower than 1%, but for the purpose of completeness, we decided to include the DNA methylation data in our statistics nevertheless. Furthermore, study participants or CpGs with more than 10% missing values were additionally excluded, leaving a total of 87 patients (cross-sectional cohort: n = 59; longitudinal cohort: n = 28) for final statistics. Methylation data of all sample types (i.e., blood, PBMCs, and defined immune cell subsets) were tested for correlation (Pearson’s correlation test) with clinical baseline parameters in the whole cohort as well as in ECT remitters/non-remitters only. Mixed linear models (residual maximum likelihood approach) were performed computing (baseline) methylation as the dependent variable. CpG position was entered as repeated measurement assuming a scaled identity of covariates. Estimated marginal means were calculated for the groups and compared by Sidak’s post-hoc test. Parameter estimates were calculated for all factors and manually inspected. Model fits were compared using the −2loglikelihood ratio. Age, gender, and BMI were considered as covariates. Results are depicted as mean ± SD (standard deviation) or ± SE (standard error). Due to the explorative character of our approach, a nominal p-value of ≤0.05 (two-tailed) has been classified as significant (and has thus been the basis for the interpretation of our results), although Type 1 error correction has only been applied to the mixed linear models. For the purpose of completeness, the adjusted p-value is additionally indicated (wherever applicable), but in the respective tables only.

## Results

### Clinical Baseline Characteristics

Baseline characteristics of patients are presented in **Table 1** (pooled cohort), and **Supplementary Material, Tables S5**, and **S6** (both cohorts separately). Prior to ECT treatment, patients were suffering from a moderate to severe depression, as the average psychometric test score was 28.5 (± 2.0; 19–34; HAM-D) and 31.7 (± 9.6; 12–45; MADRS). Among all study participants (n = 87), 33 individuals remitted upon ECT; 53 responded. 13 patients had minimally elevated levels for leukocytes (reaching values of up to 14.0 × 10^3^/μl), but no signs of infection (i.e., heightened CRP). Participants were medicated while being treated with ECT. In this regard, 28 patients were taking benzodiazepines on a regular basis, with lorazepam being (besides oxazepam, bromazepam, zolpidem, and zopiclone) one of the most commonly prescribed ones (n = 24).

**Table 1:** Clinical baseline characteristics of patients.

		Whole cohort (n = 87)	Remitters (n = 33)	Non-remitters (n = 53)
**Demographics**				
Age in years, mean (± SD; range)		51.9 (± 16.6; 20–80)	56.0 (± 14.6; 29–80)	49.7 (± 17.5; 20–80)
Gender, n (%)	female	45 (51.7%)	17 (51.5%)	27 (50.9%)
	male	42 (48.3%)	16 (48.5%)	26 (49.1%)
**Psychometric characteristics**				
Age at diagnosis in years, mean (± SD; range)		32.9 (± 15.5; 12–78)	33.9 (± 14.6; 12–65)	32.2 (± 16.3; 14–78)
Current episode in weeks, mean (± SD; range)		24.4 (± 20.8; 3–124)	19.6 (± 22.2; 3–124)	27.1 (± 19.4; 6–96)
Psychotic symptoms, n (%)	Yes	14 (16.1%)	4 (12.1%)	10 (18.9%)
**Medication**				
Antidepressant drugs, n (%)	Yes	79 (95.2%)	31 (96.9%)	47 (94.0%)
Benzodiazepines, n (%)	Yes	28 (33.7%)	12 (37.5%)	16 (32.0%)
Antipsychotic drugs, n (%)	Yes	45 (54.2%)	21 (65.6%)	23 (46.0%)
**Clinical parameters**				
Leukocytes in ×10^3^/μl, mean (± SD; range)		7.8 (± 2.3; 3.3–14.0)	7.6 (± 2.3; 3.8–12.4)	8.0 (± 2.4; 3.3–14.0)

Clinical baseline characteristics of refractory MDD patients treated with ECT (whole group vs. ECT remitters and non-remitters), presented as mean (± standard deviation (SD); range (=minimum–maximum) or quantity (absolute and percentual, n (%)). Clinical parameters were equally distributed between ECT remitters and non-remitters.

### Association of DNA Methylation With Clinical Baseline Characteristics

Analyzing the correlation between the DNA methylation and the patients’ clinical baseline characteristics (n = 87), revealed an association between the DNA methylation of total t-PA (*r* = −0.228, p = 0.033) and the MHRE region (*r* = −0.229, p = 0.033) with age. A correlation between the number of leukocytes and the mean DNA methylation of PAI-1 has further been found (*r* = −0.223, p = 0.047; **Supplementary Material, Table S7** and **Figure S2**).

### DNA Methylation in Dependence on ECT Remission

#### Blood—Baseline

**Figure 1:**
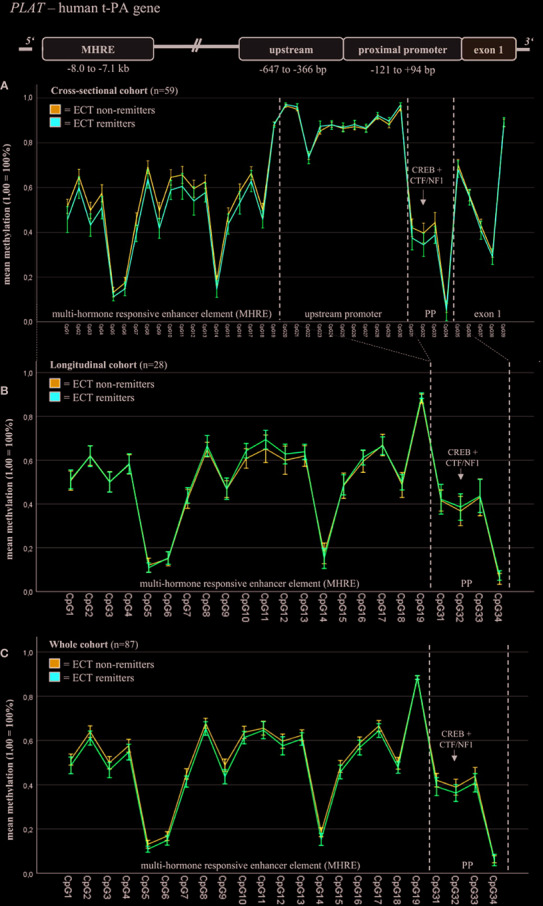
Blood baseline DNA methylation of t-PA in ECT remitters and non-remitters. Mixed linear models (including Sidak’s correction) revealed differences in the blood baseline DNA methylation of total t-PA (including fragment t-PA 2, t-PA 4, t-PA 5; see **Supplementary Material, Table S2**) and the MHRE region between ECT remitters and non-remitters in the cross-sectional cohort **(A)**; these fragments have thus been chosen for further DNA methylation analyses (**B**: longitudinal cohort, **C**: merged cohort). In this regard, DNA methylation of these fragments did not differ between ECT remitters (n = 33) and non-remitters (n = 53) when both cohorts (n = 87) were investigated **(C)**. MHRE, multi-hormone responsive enhancer; CREB, cAMP response element-binding protein; CTF/NF1, CCAAT box-binding transcription factor/nuclear factor 1; PP, proximal promoter.

Baseline DNA methylation of defined t-PA gene regions differed between ECT outcome groups in the cross-sectional cohort analyzed (**Figure 1A**); these fragments have thus been chosen for further DNA methylation analyses (**Figure 1B, C**). In this regard, DNA methylation of these fragments did not differ between ECT remitters (n = 33) and non-remitters (n = 53) when both cohorts (n = 87) were investigated (**Figure 1C**, **Supplementary Material**, **Table 2**). There were no significant DNA methylation differences within the PAI-1 gene (**Supplementary Material, Figure S3**).

**Table 2:** DNA methylation of defined t-PA and PAI-1 gene regions in ECT remitters and non-remitters.

	Baseline	Whole time course of ECT			
Region	Blood (n = 87) (R/NR (± SE), 95% CI)	Blood (n = 28) (R/NR (± SE), 95% CI)	PBMC (n = 21) (R/NR (± SE), 95% CI)	NK cells (n = 21) (R/NR (± SE), 95% CI)	T cells (n = 21) (R/NR (± SE), 95% CI)
Total t-PA	0.455/0.472 (± 0.008/0.006) 0.440–0.470/0.460–0.483	0.479/0.459(± 0.005/0.006)** 0.469–0.488/0.447–0.471	0.647/0.622(± 0.008/0.009)* 0.632–0.663/0.605–0.640	0.615/0.592(± 0.009/0.010) 0.598–0.633/0.573–0.611	0.780/0.766(± 0.007/0.008) 0.766–0.794/0.751–0.781
MHRE	0.487/0.502 (± 0.008/0.006) 0.472–0.503/0.489–0.514	0.518/0.490(± 0.005/0.007)*** 0.508–0.527/0.477–0.503	0.690/0.654(± 0.008/0.009)** 0.675–0.706/0.637–0.671	0.655/0.621(± 0.009/0.010)* 0.636–0.673/0.601–0.641	0.804/0.781(± 0.007/0.008)* 0.790–0.817/0.766–0.796
CREB	0.381/0.407 (± 0.014/0.012) 0.353–0.409/0.384–0.430	0.375/0.393(± 0.009/0.012) 0.358–0.392/0.371–0.416	0.557/0.558(± 0.016/0.018) 0.526–0.589/0.521–0.594	0.559/0.573(± 0.014/0.015) 0.531–0.587/0.543–0.603	0.835/0.847(± 0.008/0.008) 0.819–0.851/0.830–0.864
CREB + CTF/NF1	0.393/0.417 (± 0.012/0.010) 0.369–0.416/0.398–0.436	0.383/0.405(± 0.007/0.010) 0.370–0.424/0.370–0.397	0.553/0.568(± 0.013/0.015) 0.528–0.579/0.538–0.597	0.534/0.565(± 0.012/0.013) 0.512–0.557/0.540–0.590	0.801/0.824(± 0.008/0.009) 0.785–0.817/0.807–0.841
Total PAI-1	0.156/0.142 (± 0.015/0.012) 0.127–0.186/0.118–0.165	0.164/0.152(± 0.009/0.013) 0.146–0.183/0.127–0.176	Measured in blood only	Measured in blood only	Measured in blood only

The DNA methylation of defined t-PA gene regions differed between ECT remitters (R) and non-remitters (NR) in various sample types analyzed (i.e., the blood, the whole population of PBMCs (peripheral blood mononuclear cells), natural killer (NK) cells, and T cells). Results were calculated by mixed linear models (including Sidak’s correction) and are presented as mean [± standard error (SE)] and 95% confidence interval (CI). Total t-PA methylation includes three fragments (t-PA 2, t-PA 4, and t-PA 5), total PAI-1 methylation only one (PAI-1 2). For further information regarding the analyzed fragments, see **Table S2** in the Supplements. MHRE, multi-hormone responsive enhancer, CREB, cAMP response element-binding protein, CTF/NF1, CCAAT box-binding transcription factor/nuclear factor 1. *p < 0.05, **p < 0.001, ***p < 0.0001.

#### Immune Cells—Baseline

**Figure 2:**
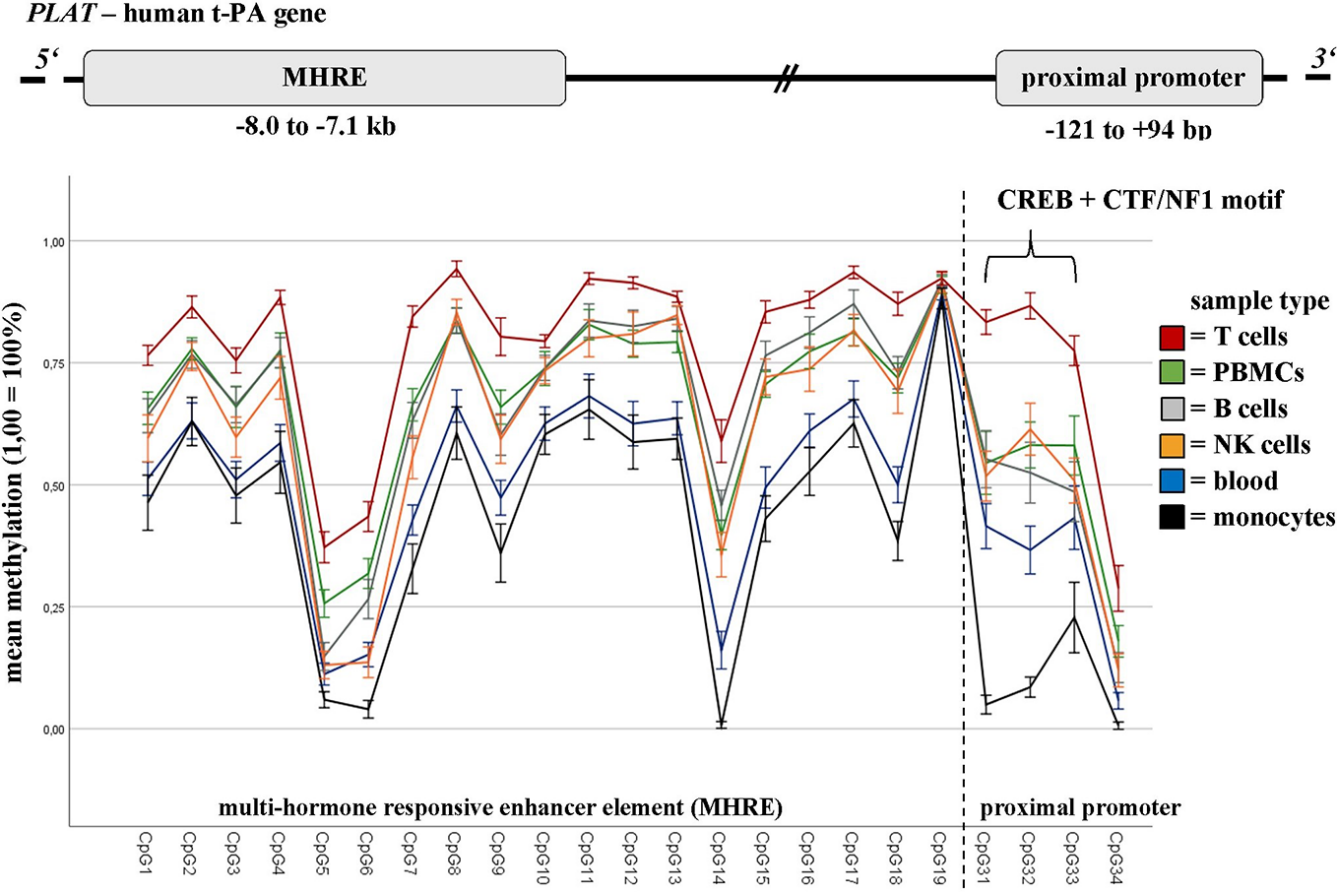
Baseline DNA methylation differences of t-PA in blood and defined immune cell subtypes. DNA methylation rates of defined t-PA gene regions (i.e., the MHRE element (CpG1–CpG18), the CREB binding site (CpG31+32) and the CTF/NF1 motif (CpG33)) differ greatly between the sample types analyzed (namely whole blood (n = 28), peripheral blood mononuclear cells (PBMCs; n = 21), and defined immune cell subsets (n = 21)), collected from refractory MDD patients undergoing a course of ECT. Results are presented as mean (1,0 = 100%), bars are indicating the standard error. MHRE, multi-hormone responsive enhancer; CREB, cAMP response element-binding protein; CTF/NF1, CCAAT box-binding transcription factor/nuclear factor 1.

The DNA methylation of defined t-PA regions showed significant differences between ECT remitters (n = 10) and non-remitters (n = 11) in NK cells, T cells, as well as in the whole population of PBMCs (but not in B cells or monocytes; see **Table 2** and **Supplementary Material, Table S8**). Major differences in t-PA’s DNA methylation were further found when the different sample types were analyzed (calculated for the whole cohort but being present also in ECT remitters and non-remitters; see **Figure 2** and **Supplementary Material, Table S9** for a detailed depiction). The highest contrast was found in the DNA methylation of the CREB binding site when T cells and monocytes were compared. These differences were present throughout the whole course of ECT as there was neither an interaction between remission nor between the sample type with the measured time points. DNA methylation of PAI-1 was measured in blood only.

#### DNA Methylation During the Course of ECT

The DNA methylation of defined t-PA gene regions changed upon a course of ECT. Regarding ECT’s acute effect (=comparing the methylation status before and after a single ECT session), DNA methylation of CREB (with or without its adjacent binding site) was found to be altered in whole blood [CREB: p = 0.030, mean methylation(± SE): before = 0.365(± 0.010), after = 0.396(± 0.010)] and in T cells (CREB+CTF/NF1: p = 0.019, before = 0.825(± 0.008), after = 0.799(± 0.008)). The latter CpGs seem to be further involved in ECT’s long-term effect: in whole blood, DNA methylation of CREB+CTF/NF1 was found to be changed following a whole course of ECT (p = 0.002), but no consistent pattern in methylation changes (in terms of a consistent acute or long-term effect) was prevalent when comparing the DNA methylation values of each ECT session. In monocytes, DNA methylation of CREB and CREB+CTF/NF1 was found to be increased after treatment completion (CREB: p = 0.028, 1^st^ ECT = 0.064(± 0.010), last ECT = 0.098(± 0.011); CREB+CTF/NF1: p = 0.023, 1^st^ ECT = 0.109(± 0.012), last ECT: 0.150(± 0.014)). Importantly, these effects seem to be independent of clinical remission (see **Supplementary Material, Tables S10** and **S11**, and **Figure S4** for more details). DNA methylation of PAI-1 (measured in blood only) was left unaffected by ECT.

## Discussion

According to our statistics, ECT remitters and non-remitters did not differ in their baseline t-PA or PAI-1 DNA methylation, rejecting thereby our hypothesis of both genes to be suitable biomarkers of ECT responsiveness. However, major differences in the DNA methylation throughout the analyzed t-PA regions were found when the different immune cell subtypes (B cells, NK cells, monocytes, and T cells) were compared. These differences remained stable throughout the whole treatment course and were independent of clinical outcome.

The pattern of DNA methylation (not to be mistaken for the methylation rate) was similar in nearly all sample types with a relatively low mean methylation in the proximal promoter region, a highly methylated upstream promoter (measured in the blood of the cross-sectional cohort only), and an MHRE element showing mean methylation values somewhere in between these two stretches. The same pattern has been reported in various other cell types [i.e., astrocytes, fibroblasts, and HUVECs (human umbilical vein endothelial cells)] (50), though its relevance for t-PA expression has not been entirely clarified yet. A study of Dunoyer-Geindre et al. (published in 2011) suggested t-PA transcription to be inhibited in case of t-PA promoter methylation, most likely due to insufficient binding of various transcription factors (i.e., CREB, CTF/NF1, and Sp1) having their motifs within this region (49, 53). The same group investigated the influence of MHRE methylation—an enhancer element that is located −7.3 kbp upstream of tPA’s first exon and that comprises several responsive units for glucocorticoids, mineralocorticoids, or androgens, for example (51)—on t-PA expression and proposed the MHRE region to be of neglectable relevance for t-PA production. Instead, they again confirmed their previous findings by showing an unmethylated proximal promoter region to be particularly required for efficient t-PA expression (50). However, transfection experiments reported the contrary and revealed the enhancer element (together with its hormonal regulators) to influence t-PA transcription, probably mediated by changes in open chromatin formation (51). This group has not investigated the effect of DNA methylation in particular, but more support for the notion of epigenetics being involved comes from Magnusson et al., who observed a decline of DNA methylation in the MHRE region to cause a concomitant increase in t-PA production in cultured human endothelial cells (52).

The inconsistency between these findings hinders a clear interpretation of the methylation pattern found in our cells, though it would suggest monocytes (among those cells analyzed) to be predominantly involved in t-PA expression. Intriguingly, monocytes had also the lowest CpG methylation at the CREB binding site—a motif repeatedly shown to be involved in depression by mediating several effects down- and upstream of BDNF (63, 64). Nonetheless, t-PA’s DNA methylation remained remarkably stable throughout the course of ECT, irrespective of the sample type analyzed, showing only marginal changes (partially with inconsistent patterns) that can be considered as negligible. Because DNA methylation was further showing only subtle differences between ECT remitters and non-remitters in sorted immune cell subpopulations, we must assume that the DNA methylation of t-PA is only of minor importance (if at all) considering ECT’s therapeutic effects. Nevertheless, one should keep in mind that we only investigated the DNA methylation of major immune cells subtypes, leaving out subsets that are quantitatively small in numbers (like T regulatory cells or non-classical monocytes, for instance). Since we observed major differences between the CpG methylation of different immune cell subtypes, we highly recommend DNA methylation analyses to be performed in defined cell subsets and not in whole blood only. Especially if we consider leukocytes to be highly adaptive and plastic (65) (at least to some extent)—changing their composition in dependence of age (66), smoking behavior (67), BMI (68), health status (69, 70), and exercise (71), for example—one must conclude whole blood DNA methylation to be influenced by hardly controllable errors, especially in smaller group sizes.

The relatively small group size is also a limitation of our pilot study. Further, the resolution of our DNA methylation data is restricted by the sequencing technique used: while Sanger sequencing is sufficient to call large methylation differences among distinct cell types (**Figure 2**), other methods (as next-generation sequencing) are preferable to capture small DNA methylation changes. Particularly the results regarding the comparison of ECT remitters/non-remitters and the different time points of ECT treatment, must be, therefore, interpreted with caution. Another circumstance troubling our approach is the medication (including the anesthetics and muscle relaxants) that the patients received while being treated with ECT. As pharmaceuticals can potentially impact DNA methylation, particularly anesthetics were further suggested to mediate antidepressant effects that are comparable to ECT treatment (72). Besides drug intake, DNA methylation changes were also associated with age (73), gender (74), and BMI (75). These three factors were, therefore, considered as covariates.

## Conclusion

Epigenetics of t-PA and PAI-1 seem to be of minor importance considering ECT’s therapeutic effects and are not suitable as biomarkers for ECT response prediction. However, t-PA’s DNA methylation differed greatly between the different immune cell subsets analyzed. This result leads to the recommendation of DNA methylation analyses to be performed in various immune cell subtypes and not in whole blood only, especially if the cohort is small and the confounding factors hardly to control.

## Data Availability Statement

The datasets generated for this study are available on request to the corresponding author.

## Ethics Statement

The study involving human participants were reviewed and approved by the Ethics Committee of the Charité (EK-224-05c) and the Hannover Medical School (2842-2015). The patients/participants provided their written informed consent to participate in this study.

## Author Contributions

NM: Establishing and conducting the experiments described in the *Material and Methods* section (excluding the clinical part). Processing data, including their analysis. Writing the paper, including the **Supplementary Material**, KJ: Providing enormous support in all experiment-related issues, MBaj: Acquisition and treatment of the cross-sectional cohort (providing clinical data and blood samples), HM and CP: Recruitment and treatment of the longitudinal cohort (providing clinical data and blood samples), MBal: Giving advice regarding flow cytometry (choice of antibodies and general procedure). Designing the layout for flow cytometry and sorting the PBMCs. AK: Support with the processing of blood, SB: Substantial contribution to the conception of the work and acquisition of patients. Revising the study for important intellectual content, HF: Substantial contribution to the conception and design of the work, the acquisition of patient groups, and the analysis and interpretation of data. Drafting the work and revising it critically for important intellectual content. AN: Supervising the study. Essential contribution to the conception and design of the work, the acquisition of patient groups, and the analysis and interpretation of data. Drafting the work and revising it for important intellectual content. Strong guidance regarding the writing of the paper, and the presentation of data.

## Funding

The research was financially supported by in-house funding from the Hannover Medical School. The grant provider had no role in study design, collection, interpretation or analysis of data nor in writing of the report or the decision to submit the paper for publication.

## Conflict of Interest

The authors declare that the research was conducted in the absence of any commercial or financial relationships that could be construed as a potential conflict of interest.

## Footnotes

1 BioLegend. Cell Surface Flow Cytometry Staining Protocol 2017: https://www.biolegend.com/protocols/cell-surface-flow-cytometry-staining-protocol/4283/ last accessed:02.10.19; 5.22 p.m.

2 Ensembl: www.ensembl.org last accessed:02.10.2019, 6.29 p.m.

3 SNPCheck V3: https://genetools.org/SNPCheck/snpcheck.htm last accessed:02.10.2019, 6.31 p.m.

4 NetPrimer: http://www.premierbiosoft.com/netprimer/ last accessed:02.10.2019, 6.33 p.m.

5 Metabion Biocalculator: http://www.metabion.com/support-and-solution/biocalculator/ last accessed:02.10.2019, 6.34 p.m.

6 Match 1.0 Public: http://gene-regulation.com/ last accessed:02.10.2019, 6.54 p.m.
