# Supplementary material for: DNA Methylation of the t-PA Gene Differs Between Various Immune Cell Subtypes Isolated From Depressed Patients Receiving Electroconvulsive Therapy
Source: Front Psychiatry. 2020 Jun 19;11:571. doi: 10.3389/fpsyt.2020.00571 (PMC7319092; doi:10.3389/fpsyt.2020.00571)
Supplement: Supplementary file 1 [file DataSheet_1.pdf]

Supplementary Material

1 Methods and Material

1.1 Processing of Patient Samples

1.1.1 PBMC Isolation

Peripheral blood mononuclear cells (PBMCs) were isolated from EDTA-blood by density centrifugation as described elsewhere (45). Following the recommendations of Mallone et al. (46), changes have been made to the latter protocol and PBMCs have been isolated as follows: 17 ml of EDTA-blood was centrifuged at 2000xg for 10 min at room temperature (RT) and 4 ml of plasma (=supernatant) removed for other purposes. The removed volume was replaced with 4 ml of PBS (Dulbecco's Phosphate Buffered Saline, w/o Ca^{2+} and Mg^{2+}; Biochrom GmbH, Berlin, Germany), and the EDTA-monovettes inverted for multiple times before transferring their content into 50 ml conical tubes (SARSTEDT AG & Co. KG, Nümbrecht, Germany). In the following, monovettes were washed twice (PBS) and further PBS added until the original blood volume was being diluted to 1:2. The diluted blood was layered onto 12 ml of Biocoll® Separating Solution (density: 1.077 g/ml; Biochrom GmbH) and the gradient subsequently centrifuged at 2000xg for 20 min (RT) with the deceleration speed set to zero (Allegra X-15R, Beckman Coulter GmbH, Krefeld, Germany). After removing the supernatant (leaving 5 ml above the buffy coat), PBMCs were pipetted into a new 50 ml tube and washed 2X with 4°C-cold PBS that was supplemented with 1% BSA (bovine serum albumin; SIGMA-ALDRICH Co., St. Louis, USA), using 1200xg for 10 min at 4°C for the centrifugation steps. Cells were then manually counted by using 0.2% trypan blue (SIGMA-ALDRICH Co.) as a viability marker and a Neubauer chamber (Paul Marienfeld GmbH & Co. KG, Lauda-Königshofen, Germany) as a counting device. After a final centrifugation (1200xg, 10 min, 4°C), fresh freezing medium (=10% human serum and 10% DMSO (dimethyl sulfoxide; SIGMA-ALDRICH Co.) in 4°C-cold PBS) was prepared to resuspend (10-20x10^6 cells/ml) and aliquot (CryoPure Tubes, SARSTEDT AG & Co. KG) the PBMCs for long-term storage. For this purpose, PBMCs (now kept in cryotubes) were put into pre-cooled (4°C) NALGENE^{TM} Cryo 1°C freezing containers (Thermo Fisher Scientific, Waltham, USA) that were filled with isopropanol (Carl Roth GmbH & Co. KG, Karlsruhe, Germany) and the container subsequently kept at -80°C. To ensure optimal preservation, PBMCs were transferred into a liquid nitrogen tank (MVE Cryosystem 750, Jutta Ohst german-cryo® GmbH, Jüchen, Germany) after 24-72 hrs.

1.1.2 Thawing of PBMCs

For the current study, PBMCs (kept in cryotubes) were thawed in a water bath (i.e., kept in 37°C until a small bit of ice remained) and 1 ml of heated RPMI 1640 medium (Biochrom GmbH; 37°C, containing 2% BSA) subsequently added to each cryotube. Cell suspensions were transferred into 50 ml conical tubes (SARSTEDT AG & Co. KG) containing 5 ml of the 2%-BSA-RPMI-medium (37°C), and 10 ml of the medium additionally added. Afterward, PBMCs were centrifuged at 900xg for 5 min at 4°C and washed twice (25 ml PBS supplemented with 1% BSA and 1mM UltraPure^{TM} EDTA (Invitrogen AG, Carlsbad, USA), referred to as FACS (fluorescence-activated cell sorting) buffer in the following). After washing, cells were put on ice for 0.5 h and manually counted by using a Neubauer chamber (Paul Marienfeld GmbH & Co. KG), 0.2% trypan blue (SIGMA-ALDRICH Co.) and an inverted CKX41 microscope (Olympus, Shinjuku, Japan). After counting, cells were separated into 5 ml polypropylene tubes (SARSTEDT AG & Co. KG) and stained as follows.

1.1.3 Staining of PBMCs

Staining was conducted as instructed by BioLegend's staining protocol[1], using 10% human serum in FACS buffer for blocking and 7-AAD (7-Amino-Actinomycin D, 1:100, BioLegend, San Diego, USA) as a viability marker. Fluorophore-conjugated antibodies were chosen based on optimal signal strength and minimal spectral overlap[2]. Titration experiments were conducted to assess optimal staining concentrations and single-stained controls were used for compensation. To prevent clumping, cells were filtered with a 35 µm nylon mesh cell strainer (attached to 5 ml Falcon®Round-Bottom tubes, Corning Life Sciences, Bedford, USA) prior to sorting.

Table S1: ***Antibodies used for flow cytometry.*** Peripheral blood mononuclear cells that were isolated from treatment-resistant depressed ECT patients were stained (and subsequently sorted) by using the antibodies listed below. 7-AAD (7-Amino-Actinomycin D, 1:100) served as a viability marker. The layout for sorting is depicted in Figure S1.

**Antibody**	**Fluorophore**	**Clone**	**Type**	**Brand**	**Dilution**	**Excitation**	**Band pass filter**
CD3	PerCP/Cy5.5	SK7	Mouse, $IgG_{1,\kappa}$	Biolegend	1/150	488 nm	695/40
CD14	AF647	HCD14	Mouse, $IgG_{1,\kappa}$	Biolegend	1/150	641 nm	670/30
CD16	BV510	3G8	Mouse, $IgG_{1,\kappa}$	Biolegend	1/125	405 nm	525/50
CD20	AF488	2H7	Mouse, $IgG_{2b,\kappa}$	Biolegend	1/250	488 nm	530/30
CD45	AF700	HI30	Mouse, $IgG_{1,\kappa}$	Biolegend	1/150	641 nm	730/45
CD56	PE	5.1H11	Mouse, $IgG_{1,\kappa}$	Biolegend	1/500	561 nm	586/15

1.1.4 Sorting of PBMCs

Immune cell populations of interest (i.e., B cells, natural killer (NK) cells, monocytes, and T cells) were sorted into 5 ml polystyrene tubes (SARSTEDT AG & Co. KG) using a BD FACSAria™ Fusion flow cytometer and the BD FACSDiva™ 8.0.1. Software (Becton, Dickinson Biosciences, Franklin Lakes, USA). Collection tubes contained a solution of 4% BSA in PBS (4°C) to optimize cell viability upon sorting. Sorted cells were centrifuged (900xg, 5 min, 4°C), the supernatant discarded, and each cell pellet resuspended in 500 µl RNAprotect Cell Reagent (QIAGEN N.V., Hilden, Germany). The samples were subsequently stored at 4°C until further use (up to 1 week). Importantly, cells were kept at 4°C and treated under sterile conditions throughout the whole process (i.e., staining, sorting, storing).

---

[1] BioLegend. Cell Surface Flow Cytometry Staining Protocol 2017: https://www.biolegend.com/protocols/cell-surface-flow-cytometry-staining-protocol/4283/ last accessed: 02.10.19; 5.22 p.m.

[2] BD Biosciences. Spectrum Viewer: https://www.bdbiosciences.com/en-us/applications/research-applications/multicolor-flow-cytometry/product-selection-tools/spectrum-viewer last accessed: 02.10.2019; 5.25 p.m.

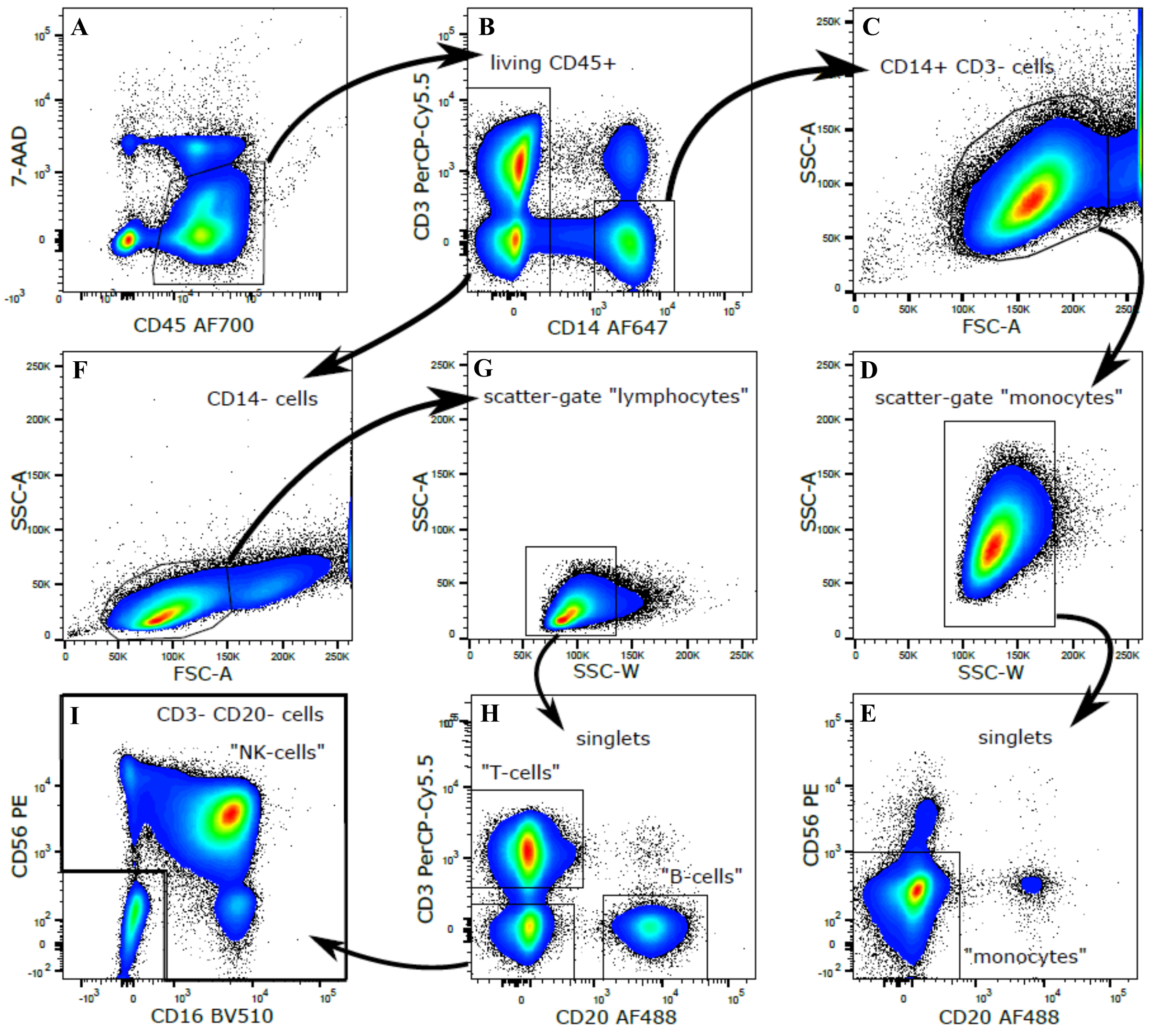

Figure S1: ***Flow cytometry layout for sorting.*** Peripheral blood mononuclear cells (isolated from depressed ECT patients) were sorted by flow cytometry to enable DNA methylation analyses in different immune cell subtypes separately. For this purpose, cells were gated as follows: **A)** red blood cells, nonviable cells as well as CD45^{-} cells were excluded using a 7-AAD/CD45 plot. **B)** Viable CD45^{+}7-AAD^{-} leukocytes were further discriminated based on their CD3 and CD14 expression. **C+F**) These cells (both CD14^{-} as well as CD14^{+}CD3^{-} cells) were then separated based on their forward (=FSC-A) and side scatter properties (=SSC-A). **D+G**) Doublets and triplets were excluded from the latter subsets via SSC-A (A=area) and SSC-W (W=width) plots, leaving only single cells for sorting. **E**) Singlets from the 'monocyte' scatter-gate were subsequently plotted via CD56/CD20 to exclude CD20^{+} B cells and CD56^{+} natural killer cells, if still prevalent. **H)** Singlets from the 'lymphocyte' scatter-gate were separated by CD3 and CD20, enabling a distinction between CD3^{+} T cells and CD20^{+} B cells. **I)** CD3^{-}CD20^{-} cells were then discriminated by their CD56 and CD16 expression, allowing an identification of natural killer cells.

1.1.5 Genomic DNA (gDNA) Isolation from PBMCs

gDNA of PBMCs and sorted immune cell subtypes was isolated using the AllPrep DNA/RNA 96 Kit (QIAGEN N.V.). Minor changes have been made to the recommended procedure. In brief, PBMCs were centrifuged at 5000xg, 4°C for 5 min before isolation. The supernatant was discarded and 300 µl of RLT buffer (containing 1% β-Mercaptoethanol (AppliChem GmbH, Darmstadt, Germany)) added to each sample. The cells were lysed by pulse vortexing (2x30 sec, 4°C) and subsequently stored on ice for 15 min minimum. The lysates were transferred into the wells of an AllPrep96 DNA plate, centrifuged (5600xg, 4 min, RT), and washed thrice (1x AW1 buffer, 2x AW2 buffer). To elute gDNA, each sample was incubated with 50 µl of heated (70°C) EB buffer for 5 min before centrifugation (4 min at 5600xg, RT). The elution was repeated, and the combined eluate kept at -80°C until further use.

Table S2: ***Primers used for PCR and sequencing.*** The following primers were used for DNA methylation analysis of bisulfite-converted DNA isolated from multiple sample types (namely blood, PBMCs, and defined immune cell subtypes) collected from treatment-resistant MDD patients undergoing a course of ECT. For DNA amplification, both primers (of a fragment) were used; for sequencing only one primer was needed. For a detailed description of the (sequencing) PCR programs used, see Tables S3 and S4. bp=base pairs, chr=chromosome, T_m=melting temperature (calculated via Metabion's Biocalculator[3]).

Region	Primer No: Sequence (5´- 3´)	T_m	Product size	Primer position in relation to exon 1	Genomic position of product	CpG No.	PCR program No. (T_m)	Sequencing PCR No.
**t-PA (tissue-type plasminogen activator)**								
t-PA 1	1: ACTTACTCCTTCCCTTTCCT	56°C	257 bp	+207 to +227	chr8:42207498 – chr8:42207754	35 – 39	1 (56.0°C)	1 (Primer 1)
	2: AGTTAGGATGGGTTGTGTTG	56°C		-30 to -10				
t-PA 2	3: AAACCATAAAAAAACTAAAACA	49°C	414 bp	+33 to +55	chr8:42207670 – chr8:42208083	31 – 34	2 (55.5°C)	2 (Primer 4)
	4: AGGTTGGTTTTGTTTTTTTA	48°C		-359 to -339				
t-PA 3	5: ATAACTCTAACCTCTCAAAAC	54°C	642 bp	-260 to -239	chr8:42207964 – chr8:42208605	20 – 23	2 (57.4°C)	1 (Primer 5)
	6: ATGATGATAGATGTTTTTGTTGA	54°C		-881 to -858				
t-PA 4	7: AACACATAAATAACACTAACAATA	53°C	401 bp	-7114 to -7090	chr8:42214816 – chr8:42215216	10 – 19	1 (56.5°C)	1 (Primer 7)
	8: AGGAGAGAGGAGTTATGGAA	56°C		-7491 to -7471				
t-PA 5	9: ACCTCCTTCCTAAATCAAACAT	56°C	652 bp	-7439 to -7417	chr8:42215143 – chr8:42215795	1 – 9	1 (58.0°C)	1 (Primer 9)
	10: TATTATTGTATTTTAGGTAGGGTGAT	58°C		-8069 to -8043				
**PAI-1 (plasminogen activator inhibitor-1)**								
PAI-1 1	11: CCTACAACCAAACACAACTA	54°C	376 bp	+16 to +36	chr7:101127124 – chr7:101126749	24 – 29	1 (53°C)	1 (Primer 11)
	12: TATTAGGGGTTTTAGGTTTTTT	53°C		-340 to -318				
PAI-1 2	13: AAAACCTAAAACCCCTAATA	50°C	283 bp	-340 to -320	chr7:101126768 – chr7:101126486	18 – 23	1 (49°C)	2 (Primer 14)
	14: TAAGAGTTTTTTGTTGGGT	49°C		-603 to -584				
PAI-1 3	15: TACTCTAAACCACCTCCAAA	54°C	449 bp	-488 to -468	chr7:101126620 – chr7:101126172	14 – 17	1 (53.0°C)	1 (Primer 15)
	16: TGTTTTTATTTTTTTTTTTTTTGTGTTT	54°C		-917 to -889				
PAI-1 4	17: ATTTCACTCACCTACCAC	51°C	566 bp	-1446 to -1428	chr7:101125660 – chr7:101125095	1 – 13	1 (51.5°C)	1 (Primer 17)
	18: ATTTTTTGGGTAGTATTTTGG	52°C		-1994 to -1973				

[3] Metabion Biocalculator: http://www.metabion.com/support-and-solution/biocalculator/ last accessed: 02.10.2019, 6.34 p.m.

Table S3: ***PCR programs.*** Two different PCR programs were used for the amplification of bisulfite-converted DNA isolated from whole blood, peripheral blood mononuclear cells, and defined immune cell subtypes, collected from refractory depressed individuals receiving ECT. Fragments of interest were amplified by using either Program 1 or 2 (see Table S2 for further information).

	**PCR Program 1 (touch-down)**		**PCR Program 2**	
**Step**	**Temperature**	**Time**	**Temperature**	**Time**
1	95°C	15 min	95°C	15 min
2	97°C	1 min	95°C	30 sec
3	95°C	30 sec	$\mathbf{T_m}$°C	1 min 30 sec
4	$\mathbf{T_m}$+10°C	45 sec	72°C	2 min 30 sec
5	68°C	1 min		
Loop	**starting at step 2 again, 15x**		**starting at step 2 again, 5x**	
6	95°C	30 sec	95°C	30 sec
7	$\mathbf{T_m}$-5°C	45 sec	$\mathbf{T_m}$°C	30 sec
8	65°C	1 min	72°C	45 sec
Loop	**starting at step 6 again, 35x**		**starting at step 6 again, 30x**	
9	65°C	5 min	72°C	4 min
10	12°C	∞	12°C	∞

Table S4: ***Sequencing PCR programs.*** For DNA methylation analysis of defined t-PA and PAI-1 regions, two different sequencing PCR approaches were established. Fragments of interest were sequenced by using either Program 1 or 2 (see Table S2 for a more detailed description).

	**Sequencing PCR 1**		**Sequencing PCR 2**	
Step	Temperature	Time	Temperature	Time
1	96°C	1 min	96°C	1 min
2	96°C	5 sec	96°C	10 sec
3	60°C	90 sec	50°C	5 sec
4	50°C	90 sec	60°C	4 min
Loop	**starting at step 2 again, 25x**		**starting at step 2 again, 28x**	
10	12°C	∞	12°C	∞

Table S5: Cross-sectional cohort: C*linical baseline characteristics.* Clinical baseline characteristics of refractory MDD patients treated with ECT (whole group vs. ECT remitters and non-remitters), presented as mean (±standard deviation (SD); range (=minimum–maximum)) or quantity (absolute and percentual, n (%)). Remitters and non-remitters differed in two parameters, namely the number of leukocytes (t-test; p=0.021, T=2.393) and the duration of the current episode (t-test; p=0.026, T=2.281). HAM-D=Hamilton Rating Scale for Depression. *p<0.05, #(adjusted) p<0.0045.

**Cross-sectional cohort**		**Whole cohort (n=59)**	**Remitters (n=21)**	**Non-remitters (n=38)**
**Demographics**				
**Age** in years, mean (±SD; range)		51.6 (±17.3; 21–80)	56.8 (±16.7; 29–80)	48.8 (±17.1; 21–80)
**Gender**, n (%)	female	28 (47.5%)	11 (52.4%)	17 (44.7%)
	male	31 (52.5%)	10 (47.6%)	21 (55.3%)
**Psychometric characteristics**				
**Age at diagnosis** in years, mean (±SD; range)		31.6 (±15.0; 12–78)	34.6 (±15.4; 12–65)	30.0 (±14.7; 14–78)
**Current episode** in weeks, mean (±SD; range)		20.4 (±12.0; 4–54)	15.8 (±9.6; 4–36)*	23.0 (±12.6; 7–54)*
**Number of episodes**, mean (±SD; range)		6.0 (±4.6; 1–20)	6.0 (±5.5; 1–20)	6.0 (±4.0; 1–20)
**HAM-D**, mean (±SD; range)		28.5 (±2.0; 19–34)	28.0 (±2.7; 19–31)	28.8 (±1.5; 27–34)
**Psychotic symptoms**, n (%)	yes	7 (11.9%)	2 (9.5%)	5 (13.2%)
**Medication**				
**Antidepressant drugs**, n (%)	yes	52 (94.5%)	19 (95.0%)	33 (94.3%)
**Benzodiazepines**, n (%)	yes	11 (20.0%)	3 (15.0%)	8 (22.9%)
**Antipsychotic drugs**, n (%)	yes	23 (41.8%)	10 (50.0%)	13 (37.1%)
**Clinical parameters**				
**Leukocytes** in x10^3/µl, mean (±SD; range)		8.1 (±2.2; 3.8–14.0)	7.1 (±2.1; 3.8–11.0)*	8.6 (±2.1; 4.3–14.0)*

Table S6: Longitudinal cohort: *Clinical baseline characteristics.* Clinical baseline characteristics of pharmaco-resistant depressed subjects undergoing a course of ECT (whole group vs. ECT remitters and non-remitters), depicted as mean (±standard deviation (SD); range (=minimum–maximum)) or quantity (absolute and percentual, n (%)). ECT remitters and non-remitters differed in their BMI (t-test; p=0.028, T=-2.329). 21 patients of this subgroup were used for DNA methylation analysis in PBMCs and sorted immune cell subtypes. BDI-II=Beck's Depression Inventory, MADRS=Montgomery-Åsberg Depression Rating Scale, MMSE=Mini-Mental State Examination, CRP=C-reactive protein. *p<0.05, #(adjusted) p<0.0029.

**Longitudinal cohort**		**Whole cohort (n=28)**	**Remitters (n=12)**	**Non-remitters (n=15)**
**Demographics**				
**Age** in years, mean (±SD; range)		52.5 (±15.5; 20–76)	54.6 (±10.4; 37–70)	51.9 (±18.9; 20–76)
**Gender**, n (%)	female	17 (60.7%)	6 (50.0%)	10 (66.7%)
	male	11 (39.3%)	6 (50.0%)	5 (33.3%)
**Body-mass-index (BMI),** mean (±SD; range)		27.7 (±6.0; 17–46)	30.7 (±6.4; 23–46)*	25.6 (±5.0; 17–35)*
**Smoking**, n (%)	yes	10 (37.0%)	6 (54.6%)	4 (26.7%)
**Psychometric characteristics**				
**Age at diagnosis** in years, mean (±SD; range)		36.0 (±16.7; 14–74)	32.4 (±13.3; 14–53)	38.4 (±19.4; 14–74)
**Current episode** in weeks, mean (±SD; range)		35.8 (±33.2; 3–124)	27.8 (±36.4; 3–124)	42.6 (±31.1; 6–96)
**BDI-II**, mean (±SD; range)		36.2 (±10.3; 16–56)	32.7 (±9.8; 16–52)	39.6 (±10.1; 17–56)
**MADRS**, mean (±SD; range)		31.7 (±9.6; 12–45)	30.3 (±12.6; 12–45)	33.4 (±7.0; 23–45)
**MMSE**, mean (±SD; range)		27.4 (±4.4; 13–30)	28.1 (±2.7; 21–30)	26.5 (±5.7; 13–30)
**Psychotic symptoms**, n (%)	yes	7 (25.0%)	2 (16.7%)	5 (33.3%)
**Suicidality**, n (%)	yes	7 (25.0%)	2 (16.7%)	5 (33.3%)
**Medication**				
**Antidepressant drugs**, n (%)	yes	27 (96.4%)	12 (100%)	14 (93.3%)
**Benzodiazepines**, n (%)	yes	17 (60.7%)	9 (75.0%)	8 (53.3%)
**Antipsychotic drugs**, n (%)	yes	22 (78.6%)	11 (91.7%)	10 (66.7%)
**Lithium**, n (%)	yes	6 (21.4%)	2 (16.7%)	4 (26.7%)
**Clinical parameters**				
**CRP**, mean (±SD; range)		2.6 (±2.5; 0.3–8.7)	3.1 (±3.4; 0.4–8.7)	2.4 (±2.1; 0.3–5.5)
**Leukocytes** in x10^3/µl, mean (±SD; range)		7.2 (±2.6; 3.3–12.4)	8.3 (±2.5; 5.0–12.4)	6.6 (±2.3; 3.3–12.1)

Table S7: Whole cohort: *Association of clinical baseline characteristics with the DNA methylation of defined gene regions.* According to our statistics, DNA methylation of t-PA (=t-PA 2, 4, and 5; see Table S2) as well as the multi-hormone responsive enhancer (MHRE) element correlated with the patients' age. Further, the number of leukocytes was found to be associated with the DNA methylation of PAI-1 (=fragment PAI-1 2; see Table S2). CREB=cAMP response element-binding protein, CTF/NF1=CCAAT box-binding transcription factor/nuclear factor 1, *r*=Pearson's correlation coefficient, T=T value (t-test),*p<0.05, #(adjusted) p<0.0011.

**Whole Cohort (n=87)**	Target regions				
Clinical Baseline Characteristics	**t-PA**	**MHRE**	**CREB**	**CREB + CTF/NF1**	**PAI-1**
**Demographics**					
Age [*r*]	-0.228*	-0.229*	-0.063	-0.118	-0.055
Gender [T]	0.612	0.746	-0.564	-0.367	1.331
**Psychometric characteristics**					
Age at diagnosis [*r*]	-0.153	-0.157	-0.056	-0.037	0.001
Current episode [*r*]	-0.055	-0.071	0.133	0.069	0.029
Psychotic symptoms [T]	0.549	0.732	0.296	0.417	1.509
**Medication**					
Antidepressant drugs [T]	0.777	1.258	-1.372	-1.304	1.096
Benzodiazepines [T]	0.168	0.368	-1.212	-1.283	-1.387
Antipsychotic drugs [T]	1.658	1.583	0.473	0.865	-1.150
**Clinical parameters**					
Leukocytes [*r*]	-0.063	-0.097	-0.092	-0.060	-0.223*

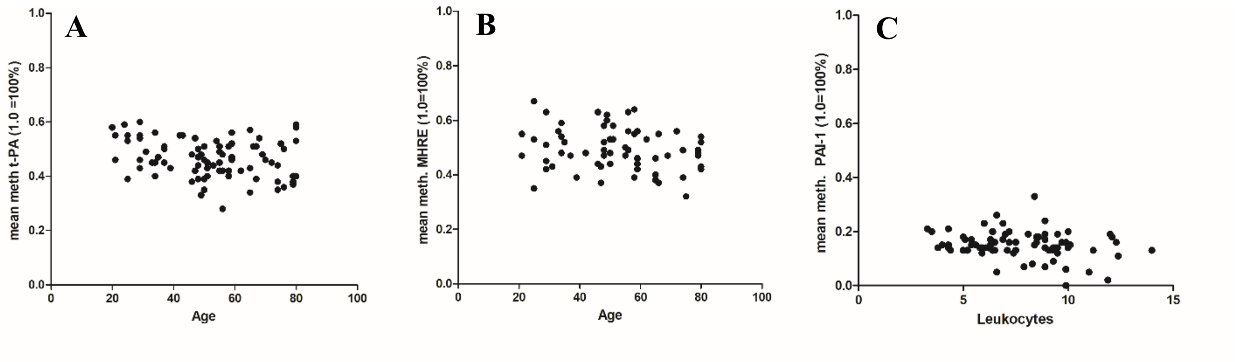

Figure S2: Pooled cohort: *Association of clinical baseline characteristics with the DNA methylation of defined gene regions.* According to our statistics, DNA methylation of t-PA (**A**) as well as the multi-hormone responsive enhancer (MHRE; **B**) element correlated with the patients' age. Further, the number of leukocytes was found to be associated with the DNA methylation of PAI-1 (**C**).

Table S8: ***DNA methylation of defined t-PA regions in ECT remitters and non-remitters.*** The DNA methylation of defined t-PA regions differs between ECT remitters (R) and non-remitters (NR) in various sample types analyzed, but not in B cells or monocytes. Sample types that showed significant results are presented in Table 2, the table below shows non-significant results of B cells and monocytes only. Statistics were calculated by mixed linear models (including Sidak's correction) and are presented as mean (±standard error (SE)) and 95% confidence interval (95% CI). Total t-PA methylation includes three fragments: t-PA 2, t-PA 4, and t-PA 5 (see Table S2 for further details). MHRE=multi-hormone responsive enhancer, CREB=cAMP response element-binding protein, CTF/NF1=CCAAT box-binding transcription factor/nuclear factor 1.

	**Whole time course of ECT**			
Sample type	**B cells**<br>[R/NR (±SE), 95% CI]		**Monocytes**<br>[R/NR (±SE), 95% CI]	
**t-PA methylation**				
Total	0.632/0.636(±0.009/0.009)<br>0.615–0.649/0.618–0.654	**R=NR**	0.401/0.376(±0.009/0.010)<br>0.382–0.419/0.356–0.396	**R=NR**
MHRE	0.679/0.677(±0.009/0.009)<br>0.662–0.696/0.659–0.696	**R=NR**	0.464/0.436(±0.009/0.010)<br>0.446–0.482/0.416–0.455	**R=NR**
CREB	0.529/0.546(±0.018/0.019)<br>0.493–0.564/0.508–0.585	**R=NR**	0.083/0.079(±0.011/0.012)<br>0.061–0.105/0.055–0.102	**R=NR**
CREB + CTF/NF1	0.509/0.550(±0.014/0.015)<br>0.482–0.536/0.520–0.580	**R=NR**	0.132/0.128(±0.013/0.014)<br>0.106–0.158/0.099–0.156	**R=NR**

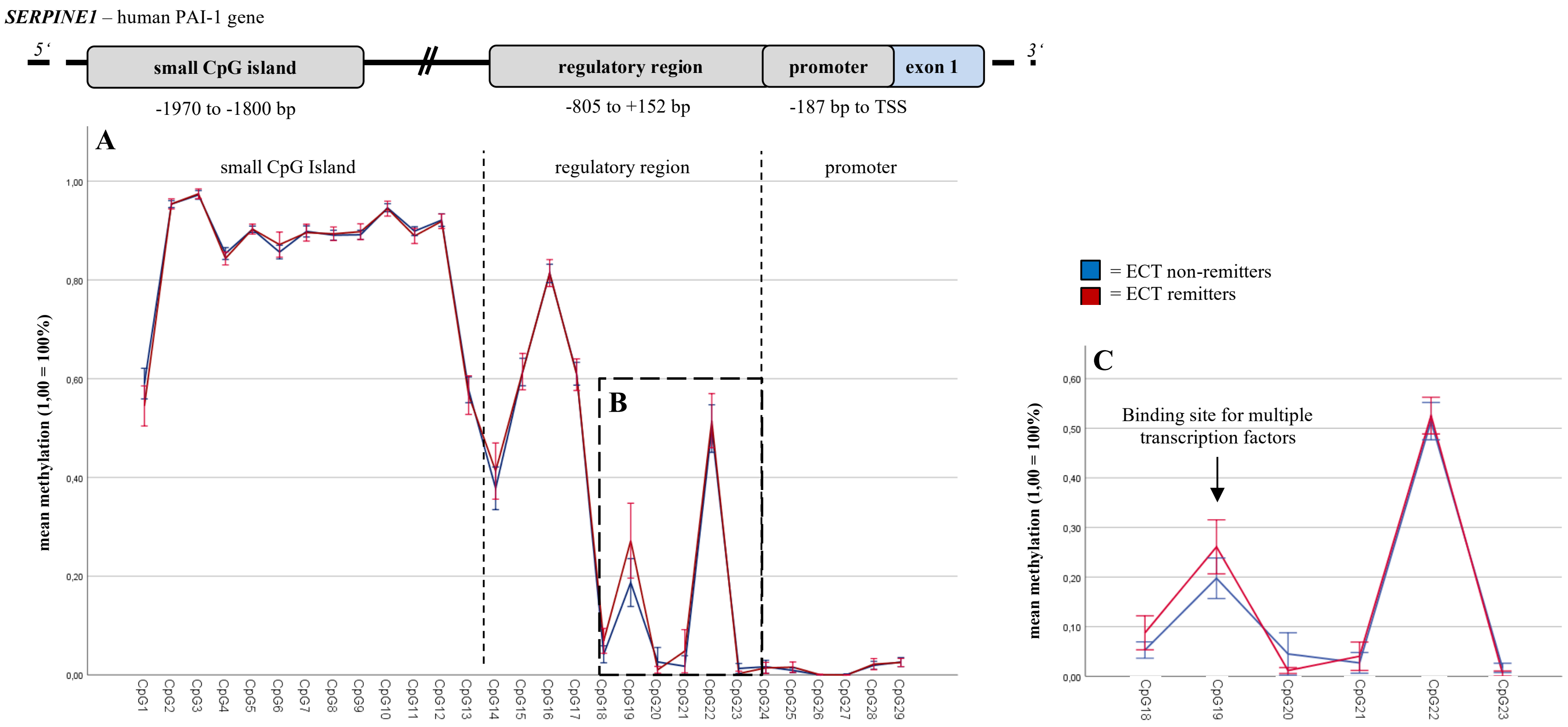

Figure S3: ***DNA methylation of PAI-1.*** The inter-individual DNA methylation variance of almost all CpGs within the plasminogen activator inhibitor-1 (PAI-1) gene was below 1%. An example of its methylation pattern (showing non-significant differences between ECT remitters and non-remitters of the cross-sectional cohort, n=59 (**A**)) is depicted in the figure above. To obtain a larger group size for subsequent DNA methylation analyses, one interesting fragment (=binding site for multiple transcription factors (**B**)) was repeatedly sequenced in the blood samples of the longitudinal cohort nevertheless. **C** is showing the baseline difference between ECT remitters/non-remitters in the whole cohort (n=87) of refractory MDD patients. TSS=transcriptional start site.

Table S9: ***DNA methylation differences in t-PA between the sample types analyzed.*** Major differences in DNA methylation were found in all regions when the different sample types were compared (mixed linear models, Sidak's correction). Results are presented as the difference between the mean values of sample type A and B. The standard error (SE) and confidence interval (95% CI) are additionally given. t-PA= tissue-type plasminogen activator, MHRE=multi-hormone responsive enhancer, CREB=cAMP response element-binding protein, CTF/NF1=CCAAT box-binding transcription factor/nuclear factor 1. *p<0.05, **p<0.001, ***p<0.0001, ****p<0.00001.

Sample type (A)	Sample type (B)	**t-PA** [mean diff. A–B (±SE), 95% CI]	**MHRE** [mean diff. A–B (±SE), 95% CI]	**CREB** [mean diff. A–B (±SE), 95% CI]	**CREB+CTF/NF1** [mean diff. A–B (±SE), 95% CI]
**Blood**	**PBMC**	-0.166(±0.008), -0.190–(-0.141)****	-0.169(±0.008), -0.194–(-0.145)****	-0.160(±0.014), -0.202–(-0.119)****	-0.154(±0.012), -0.190–(-0.118)****
	**B cells**	-0.163(±0.008), -0.187–(-0.139)****	-0.174(±0.008), -0.198–(-0.150)****	-0.140(±0.014), -0.180–(-0.099)****	-0.123(±0.012), -0.158–(-0.088)****
	**NK cells**	-0.133(±0.008), -0.157–(-0.110)****	-0.134(±0.008), -0.158–(-0.110)****	-0.169(±0.014), -0.209–(-0.128)****	-0.144(±0.012), -0.179–(-0.109)****
	**Monocytes**	0.082(±0.008), 0.058–0.106****	0.054(±0.008), 0.030–0.078)****	0.316(±0.014), 0.275–0.357****	0.276(±0.012), 0.240–0.311****
	**T cells**	-0.303(±0.008), -0.327–(-0.279)****	-0.289(±0.008), -0.313–(-0.264)****	-0.444(±0.014), -0.485–(-0.404)****	-0.406(±0.012), -0.441–(-0.371)****
**PBMC**	**Blood**	0.166(±0.008), 0.141–0.190****	0.169(±0.008), 0.145–0.194****	0.160(±0.014), 0.119–0.202****	0.154(±0.012), 0.118–0.190****
	**B cells**	0.002(±0.008), -0.022–0.026	-0.005(±0.008), -0.029–0.020	0.021(±0.014), -0.020–0.062	0.031(±0.012), -0.004–0.067
	**NK cells**	0.032(±0.008), 0.008–0.056**	0.035(±0.008), 0.011–0.060***	-0.008(±0.014), -0.050–0.033	0.010(±0.012), -0.026–0.046
	**Monocytes**	0.247(±0.008), 0.223–0.271****	0.223(±0.008), 0.199–0.248****	0.476(±0.014), 0.435–0.518****	0.430(±0.012), 0.394–0.466****
	**T cells**	-0.137(±0.008), -0.161–(-0.113)****	-0.119(±0.008), -0.144–(-0.095)****	-0.284(±0.014), -0.325–(-0.242)****	-0.252(±0.012), -0.288–(-0.216)****
**B cells**	**Blood**	0.163(±0.008), 0.139–0.187****	0.174(±0.008), 0.150–0.198****	0.140(±0.014), 0.099–0.180****	0.123(±0.012), 0.088–0.158****
	**PBMC**	-0.002(±0.008), -0.026–0.022	0.005(±0.008), -0.020–0.029	-0.021(±0.014), -0.062–0.020	-0.031(±0.012), -0.067–0.004
	**NK cells**	0.030(±0.008), 0.006–0.053**	0.040(±0.008), 0.016–0.064****	-0.029(±0.014), -0.069–0.011	-0.021(±0.012), -0.056–0.013
	**Monocytes**	0.245(±0.008), 0.222–0.269****	0.228(±0.008), 0.204–0.252****	0.455(±0.014), 0.415–0.496****	0.399(±0.012), 0.364–0.433****
	**T cells**	-0.139(±0.008), -0.163–(-0.116)****	-0.114(±0.008), -0.138–(-0.091)****	-0.305(±0.014), -0.345–(-0.265)****	-0.283(±0.012), -0.318–(-0.249)****
**NK cells**	**Blood**	0.133(±0.008), 0.110–0.157****	0.134(±0.008), 0.110–0.158****	0.169(±0.014), 0.128–0.209****	0.144(±0.012), 0.109–0.179****
	**PBMC**	-0.032(±0.008), -0.056–(-0.008)**	-0.035(±0.008), -0.060–(-0.011)***	0.008(±0.014), -0.033–0.050	-0.010(±0.012), -0.046–0.026
	**B cells**	-0.030(±0.008), -0.053–(-0.006)**	-0.040(±0.008), -0.064–(-0.016)****	0.029(±0.014), -0.011–0.069	0.021(±0.012), -0.013–0.056
	**Monocytes**	0.215(±0.008), 0.192–0.239****	0.188(±0.008), 0.164–0.212****	0.485(±0.014), 0.444–0.525****	0.420(±0.012), 0.385–0.455****
	**T cells**	-0.169(±0.008), -0.193–(-0.146)****	-0.154(±0.008), -0.178–(-0.130)****	-0.275(±0.014), -0.316–(-0.235)****	-0.262(±0.012), -0.297–(-0.227)****
**Monocytes**	**Blood**	-0.082(±0.008), -0.106–(-0.058)****	-0.054(±0.008), -0.078–(-0.030)****	-0.316(±0.014), -0.357–(-0.275)****	-0.276(±0.012), -0.311–(-0.240)****
	**PBMC**	-0.247(±0.008), -0.271–(-0.223)****	-0.223(±0.008), -0.248–(-0.199)****	-0.476(±0.014), -0.518–(-0.435)****	-0.430(±0.012), -0.466–(-0.394)****
	**B cells**	-0.245(±0.008), -0.269–(-0.222)****	-0.228(±0.008), -0.252–(-0.204)****	-0.455(±0.014), -0.496–(-0.415)****	-0.399(±0.012), -0.433–(-0.364)****
	**NK cells**	-0.215(±0.008), -0.239–(-0.192)****	-0.188(±0.008), -0.212–(-0.164)****	-0.485(±0.014), -0.525–(-0.444)****	-0.420(±0.012), -0.455–(-0.385)****
	**T cells**	-0.384(±0.008), -0.408–(-0.361)****	-0.342(±0.008), -0.366–(-0.318)****	-0.760(±0.014), -0.801–(-0.720)****	-0.682(±0.012), -0.717–(-0.647)****
**T cells**	**Blood**	0.303(±0.008), 0.279–0.327****	0.289(±0.008), 0.264–0.313****	0.444(±0.014), 0.404–0.485****	0.406(±0.012), 0.371–0.441****
	**PBMC**	0.137(±0.008), 0.113–0.161****	0.119(±0.008), 0.095–0.144****	0.284(±0.014), 0.242–0.325****	0.252(±0.012), 0.216–0.288****
	**B cells**	0.139(±0.008), 0.116–0.163****	0.114(±0.008), 0.091–0.138****	0.305(±0.014), 0.265–0.345****	0.283(±0.012), 0.249–0.318****
	**NK cells**	0.169(±0.008), 0.146–0.193****	0.154(±0.008), 0.130–0.178****	0.275(±0.014), 0.235–0.316****	0.262(±0.012), 0.227–0.297****
	**Monocytes**	0.384(±0.008), 0.361–0.408****	0.342(±0.008), 0.318–0.366****	0.760(±0.014), 0.720–0.801****	0.682(±0.012), 0.647–0.717****

Table S10: ***DNA methylation changes during the time course of ECT.*** The DNA methylation of defined t-PA gene regions changed upon a course of ECT (see Table S11). The current table is presenting the values of each time point in different subtypes, separated by ECT remitters (R) and non-remitters (NR). Results were calculated via mixed linear models and corrected with Sidak's post-hoc test. They are presented as mean (±standard error (SE)) and the 95% confidence interval (CI) is additionally given. MHRE=multi-hormone responsive enhancer, CREB=cAMP response element-binding protein, CTF/NF1=CCAAT box-binding transcription factor/nuclear factor 1, W=whole cohort, t1=before 1st ECT, t2=after 1st ECT, t3=before 4th ECT, t4=after 4th ECT, t5=before last ECT, t6=after last ECT, t7=before 1st maintenance ECT, t8=after 1st maintenance ECT. ^^=groups of remitters and non-remitters were too small to calculate the respective values. *p<0.05, **p<0.001, ***p<0.0001, ****p<0.00001.

	**Total t-PA**<br>(W/R/NR (±SE), 95% CI)	**MHRE**<br>(W/R/NR (±SE), 95% CI)	**CREB**<br>(W/R/NR (±SE), 95% CI)	**CREB + CTF/NF1**<br>(W/R/NR (±SE), 95% CI)
**Blood (n=28)**				
t1	0.469/0.476/0.463 (±0.008/0.013/0.011),<br>0.453–0.486/0.451–0.500/0.441–0.486	0.501/0.508/0.493 (±0.009/0.013/0.012),<br>0.484–0.518/0.483–0.534/0.470–0.517	0.404/0.404/0.404 (±0.016/0.023/0.021),<br>0.373–0.434/0.358–0.449/0.363–0.446	0.414/0.413/0.416 (±0.013/0.019/0.017),<br>0.390–0.439/0.375–0.450/0.382–0.450
t2	0.471/0.490/0.452 (±0.008/0.013/0.011),<br>0.454–0.488/0.465–0.515/0.430–0.475	0.506/0.530/0.482 (±0.009/0.013/0.012),<br>0.488–0.523/0.503–0.556/0.459–0.505	0.393/0.389/0.397 (±0.016/0.023/0.021),<br>0.363–0.424/0.344–0.435/0.355–0.438	0.403/0.406/0.400 (±0.013/0.019/0.017),<br>0.378–0.428/0.368–0.443/0.366–0.434
t3	0.462/0.480/0.445 (±0.009/0.014/0.013),<br>0.444–0.481/0.453–0.507/0.419–0.471	0.503/0.526/0.480 (±0.010/0.014/0.014),<br>0.484–0.522/0.497–0.554/0.454–0.507	0.351/0.330/0.372 (±0.017/0.025/0.024),<br>0.317–0.385/0.280–0.380/0.326–0.419	0.358/0.346/0.370 (±0.014/0.021/0.020),<br>0.330–0.385/0.305–0.386/0.331–0.408
t4	0.469/0.477/0.461 (±0.010/0.015/0.013),<br>0.450–0.488/0.448–0.505/0.435–0.486	0.507/0.519/0.495 (±0.010/0.015/0.013),<br>0.487–0.526/0.490–0.549/0.469–0.521	0.375/0.373/0.377 (±0.018/0.027/0.024),<br>0.340–0.410/0.321–0.426/0.330–0.423	0.376/0.370/0.382 (±0.015/0.022/0.020),<br>0.347–0.405/0.327–0.413/0.344–0.421
t5	0.465/0.480/0.450 (±0.011/0.013/0.017),<br>0.444–0.486/0.455–0.506/0.417–0.482	0.499/0.519/0.479 (±0.011/0.014/0.017),<br>0.478–0.520/0.492–0.546/0.445–0.513	0.384/0.374/0.394 (±0.019/0.024/0.030),<br>0.346–0.422/0.326–0.422/0.335–0.454	0.395/0.390/0.400 (±0.016/0.020/0.026),<br>0.363–0.426/0.350–0.429/0.350–0.450
t6	0.472/0.493/0.452 (±0.011/0.013/0.017),<br>0.452–0.493/0.467–0.519/0.419–0.484	0.502/0.528/0.475 (±0.011/0.014/0.017),<br>0.480–0.523/0.501–0.555/0.442–0.509	0.428/0.446/0.410 (±0.019/0.024/0.030),<br>0.390–0.466/0.398–0.494/0.350–0.470	0.430/0.429/0.431 (±0.016/0.020/0.026),<br>0.398–0.461/0.390–0.468/0.381–0.481
t7	0.477/0.476/0.478 (±0.015/0.015/0.025),<br>0.448–0.506/0.445–0.506/0.429–0.527	0.514/0.517/0.510 (±0.015/0.016/0.026),<br>0.484–0.544/0.485–0.549/0.459–0.561	0.368/0.339/0.396 (±0.027/0.029/0.046),<br>0.314–0.421/0.283–0.395/0.305–0.487	0.390/0.356/0.425 (±0.022/0.023/0.038),<br>0.346–0.434/0.310–0.401/0.351–0.499
t8	0.479/0.485/0.472 (±0.015/0.016/0.025),<br>0.449–0.508/0.454–0.516/0.423–0.522	0.512/0.521/0.503 (±0.015/0.017/0.026),<br>0.481–0.542/0.488–0.553/0.452–0.554	0.402/0.407/0.396 (±0.027/0.029/0.046),<br>0.348–0.455/0.351–0.463/0.305–0.487	0.416/0.418/0.414 (±0.022/0.023/0.038),<br>0.372–0.460/0.372–0.464/0.339–0.488
t9	0.456 (±0.014)^^<br>0.429–0.483	0.501 (±0.014)^^<br>0.472–0.529	0.313 (±0.025)^^<br>0.263–0.363	0.326 (±0.021)^^<br>0.285–0.367
t10	0.473 (±0.014)^^<br>0.446–0.500	0.511 (±0.014)^^<br>0.483–0.540	0.376 (±0.025)^^<br>0.326–0.426	0.382 (±0.021)^^<br>0.341–0.423

**PBMCs (n=21)**				
t1	0.637/0.653/0.621 (±0.010/0.014/0.014), 0.618–0.655/0.625–0.680/0.593–0.648	0.673/0.691/0.655 (±0.010/0.014/0.014), 0.654–0.692/0.664–0.718/0.628–0.683	0.564/0.587/0.541 (±0.019/0.028/0.028), 0.526–0.602/0.532–0.642/0.486–0.596	0.570/0.580/0.561 (±0.015/0.023/0.023), 0.540–0.601/0.535–0.624/0.516–0.605
t2	0.635/0.643/0.627 (±0.010/0.014/0.014), 0.616–0.654/0.616–0.671/0.599–0.654	0.670/0.684/0.657 (±0.010/0.014/0.014), 0.652–0.689/0.657–0.711/0.630–0.684	0.580/0.571/0.589 (±0.019/0.028/0.028), 0.543–0.618/0.516–0.626/0.534–0.645	0.577/0.561/0.593 (±0.015/0.023/0.023), 0.547–0.608/0.517–0.606/0.548–0.637
t5	0.633/0.649/0.617 (±0.013/0.016/0.020), 0.608–0.657/0.618–0.680/0.578–0.656	0.669/0.693/0.646 (±0.012/0.016/0.019), 0.645–0.694/0.662–0.724/0.607–0.684	0.547/0.526/0.569 (±0.025/0.032/0.039), 0.498–0.597/0.463–0.588/0.491–0.646	0.558/0.540/0.577 (±0.021/0.026/0.033), 0.517–0.599/0.489–0.590/0.512–0.641
t6	0.635/0.645/0.625 (±0.013/0.016/0.020), 0.610–0.660/0.614–0.676/0.586–0.664	0.675/0.692/0.657 (±0.012/0.016/0.019), 0.650–0.699/0.661–0.723/0.619–0.695	0.538/0.546/0.531 (±0.027/0.032/0.044), 0.485–0.592/0.483–0.608/0.444–0.619	0.536/0.532/0.541 (±0.022/0.026/0.036), 0.493–0.579/0.481–0.582/0.470–0.611
**B cells (n=21)**				
t1	0.631/0.625/0.636 (±0.011/0.016/0.016), 0.609–0.652/0.594–0.656/0.605–0.666	0.677/0.672/0.683 (±0.011/0.016/0.016), 0.656–0.698/0.641–0.703/0.652–0.713	0.537/0.547/0.528 (±0.022/0.033/0.032), 0.494–0.581/0.482–0.611/0.465–0.591	0.520/0.515/0.524 (±0.017/0.025/0.025), 0.486–0.553/0.466–0.565/0.475–0.572
t2	0.634/0.631/0.636 (±0.011/0.016/0.016), 0.612–0.655/0.600–0.662/0.605–0.666	0.682/0.684/0.681 (±0.011/0.016/0.016), 0.661–0.703/0.653–0.715/0.650–0.711	0.521/0.512/0.529 (±0.022/0.033/0.032), 0.477–0.564/0.447–0.576/0.466–0.593	0.507/0.494/0.520 (±0.017/0.025/0.025), 0.473–0.541/0.444–0.543/0.472–0.569
t5	0.637/0.629/0.644 (±0.013/0.017/0.020), 0.611–0.662/0.596–0.662/0.604–0.684	0.680/0.679/0.680 (±0.013/0.017/0.020), 0.654–0.705/0.646–0.712/0.640–0.720	0.532/0.476/0.589 (±0.027/0.034/0.042), 0.479–0.585/0.408–0.544/0.507–0.671	0.534/0.471/0.598 (±0.021/0.027/0.033), 0.493–0.576/0.418–0.524/0.533–0.663
t6	0.635/0.641/0.628 (±0.013/0.017/0.020), 0.609–0.660/0.608–0.674/0.589–0.668	0.673/0.680/0.667 (±0.013/0.017/0.020), 0.648–0.699/0.647–0.713/0.627–0.706	0.560/0.580/0.540 (±0.027/0.034/0.042), 0.507–0.612/0.511–0.648/0.457–0.622	0.556/0.555/0.558 (±0.021/0.027/0.033), 0.515–0.598/0.503–0.608/0.493–0.623
**Monocytes (n=21)**				
t1	0.384/0.391/0.377 (±0.012/0.017/0.017), 0.361–0.407/0.357–0.425/0.344–0.410	0.446/0.453/0.440 (±0.011/0.017/0.017), 0.424–0.469/0.420–0.486/0.407–0.472	0.064/0.061/0.066 (±0.014/0.021/0.020), 0.037–0.091/0.020–0.103/0.028–0.105	0.115/0.107/0.122 (±0.016/0.025/0.024), 0.082–0.147/0.058–0.156/0.076–0.169
t2	0.392/0.400/0.384 (±0.012/0.017/0.017), 0.369–0.415/0.366–0.434/0.351–0.417	0.455/0.463/0.446 (±0.011/0.017/0.017), 0.432–0.477/0.430–0.496/0.414–0.479	0.065/0.064/0.065 (±0.014/0.021/0.020), 0.037–0.092/0.022–0.105/0.025–0.105	0.104/0.112/0.095 (±0.017/0.025/0.024), 0.071–0.137/0.064–0.161/0.047–0.143
t5	0.395/0.406/0.384 (±0.014/0.018/0.022), 0.367–0.422/0.370–0.441/0.341–0.426	0.454/0.469/0.439 (±0.014/0.018/0.021), 0.427–0.481/0.434–0.504/0.397–0.482	0.114/0.109/0.119 (±0.016/0.021/0.025), 0.082–0.146/0.068–0.151/0.070–0.169	0.166/0.157/0.175 (±0.019/0.025/0.030), 0.129–0.204/0.109–0.206/0.117–0.234
t6	0.383/0.406/0.360 (±0.014/0.018/0.022), 0.355–0.410/0.371–0.441/0.317–0.402	0.443/0.470/0.417 (±0.014/0.018/0.021), 0.416–0.471/0.435–0.505/0.375–0.459	0.081/0.098/0.064 (±0.016/0.021/0.025), 0.049–0.113/0.057–0.139/0.015–0.114	0.135/0.151/0.118 (±0.019/0.025/0.031), 0.096–0.173/0.103–0.200/0.058–0.178
**NK cells (n=21)**				
t1	0.601/0.615/0.587 (±0.011/0.016/0.016), 0.579–0.623/0.583–0.647/0.556–0.618	0.637/0.653/0.620 (±0.012/0.017/0.017), 0.614–0.659/0.619–0.687/0.587–0.653	0.567/0.576/0.559 (±0.017/0.025/0.025), 0.533–0.601/0.526–0.626/0.509–0.608	0.549/0.549/0.548 (±0.014/0.021/0.021), 0.521–0.576/0.508–0.590/0.508–0.589
t2	0.600/0.608/0.592 (±0.011/0.016/0.016), 0.578–0.622/0.576–0.640/0.561–0.623	0.636/0.647/0.625 (±0.012/0.017/0.017), 0.613–0.659/0.614–0.681/0.592–0.658	0.548/0.542/0.555 (±0.018/0.027/0.025), 0.513–0.583/0.489–0.595/0.505–0.604	0.532/0.516/0.548 (±0.015/0.022/0.021), 0.503–0.561/0.473–0.560/0.507–0.589

t5	0.611/0.630/0.592 (±0.013/0.017/0.021), 0.585–0.637/0.596–0.663/0.551–0.632	0.646/0.672/0.620 (±0.014/0.018/0.022), 0.618–0.673/0.636–0.707/0.577–0.663	0.557/0.558/0.556 (±0.021/0.027/0.032), 0.516–0.598/0.504–0.611/0.492–0.620	0.547/0.534/0.559 (±0.017/0.022/0.027), 0.513–0.581/0.491–0.578/0.507–0.612
t6	0.602/0.608/0.596 (±0.013/0.017/0.021), 0.576–0.628/0.574–0.641/0.556–0.637	0.632/0.647/0.617 (±0.014/0.018/0.022), 0.605–0.660/0.612–0.683/0.574–0.660	0.591/0.560/0.621 (±0.021/0.027/0.032), 0.550–0.632/0.507–0.613/0.557–0.685	0.572/0.538/0.606 (±0.017/0.022/0.027), 0.538–0.606/0.495–0.582/0.552–0.660
**T cells (n=21)**				
t1	0.776/0.785/0.768 (±0.009/0.013/0.013), 0.759–0.794/0.760–0.810/0.743–0.793	0.795/0.808/0.781 (±0.009/0.013/0.013), 0.778–0.812/0.783–0.833/0.757–0.806	0.853/0.847/0.859 (±0.010/0.014/0.014), 0.833–0.872/0.818–0.875/0.831–0.887	0.827/0.818/0.837 (±0.010/0.015/0.014), 0.808–0.847/0.789–0.846/0.809–0.865
t2	0.768/0.774/0.761 (±0.009/0.013/0.013), 0.751–0.785/0.749–0.800/0.737–0.786	0.787/0.801/0.772 (±0.009/0.013/0.013), 0.769–0.804/0.776–0.826/0.748–0.797	0.842/0.826/0.859 (±0.010/0.015/0.014), 0.823–0.862/0.796–0.856/0.831–0.887	0.808/0.782/0.835 (±0.010/0.015/0.014), 0.788–0.828/0.752–0.812/0.807–0.863
t5	0.774/0.779/0.768 (±0.011/0.014/0.016), 0.753–0.795/0.753–0.806/0.736–0.800	0.792/0.801/0.782 (±0.010/0.014/0.016), 0.771–0.812/0.775–0.828/0.750–0.814	0.851/0.841/0.861 (±0.012/0.015/0.018), 0.828–0.874/0.811–0.871/0.825–0.897	0.823/0.809/0.838 (±0.012/0.015/0.019), 0.800–0.847/0.778–0.839/0.801–0.874
t6	0.774/0.782/0.767 (±0.011/0.014/0.016), 0.753–0.795/0.755–0.809/0.734–0.799	0.796/0.804/0.788 (±0.010/0.014/0.016), 0.775–0.817/0.777–0.831/0.756–0.820	0.818/0.828/0.809 (±0.012/0.016/0.018), 0.795–0.842/0.796–0.859/0.773–0.845	0.790/0.796/0.784 (±0.012/0.016/0.019), 0.766–0.814/0.764–0.828/0.748–0.821

Table S11: ***Acute and long-term ECT-associated DNA methylation changes.*** Mixed linear models (corrected by Sidak's post-hoc test) revealed acute (=difference between before and after a single ECT session) as well as long-term (=difference between before the 1st and last ECT) DNA methylation changes in defined gene regions encoding for the tissue-type plasminogen activator (t-PA) in different sample types. Results are presented as mean (±standard error (SE)); the 95% confidence interval (95% CI) is additionally given. MHRE=multi-hormone responsive enhancer, CREB=cAMP response element-binding protein, CTF/NF1=CCAAT box-binding transcription factor/nuclear factor 1. *p<0.05, **p<0.001.

Whole cohort	Total t-PA	MHRE	CREB	CREB + CTF/NF1
**Blood (n=28)**				
**Acute effect** (before/after ECT(±SE), 95% CI)	0.467/0.473 (±0.005/0.005), 0.457–0.477/0.463–0.483	0.504/0.507 (±0.005/0.005), 0.493–0.514/0.497–0.518	0.365/0.396 (±0.010/0.010), 0.351–0.388/0.378–0.415*	0.382/0.403 (±0.008/0.008), 0.367–0.397/0.388–0.419
**Long-term effect** (1st/last main. ECT(±SE), 95% CI)	0.470/0.465 (±0.006/0.010), 0.458–0.482/0.446–0.484	0.503/0.506 (±0.006/0.010), 0.491–0.516/0.486–0.526	0.399/0.344 (±0.011/0.018), 0.377–0.420/0.308–0.380	0.409/0.354 (±0.009/0.015), 0.391–0.426/0.325–0.383**
**PBMCs (n=21)**				
**Acute effect** (before/after ECT(±SE), 95% CI)	0.635/0.635 (±0.008/0.008), 0.619–0.650/0.620–0.650	0.671/0.673 (±0.008/0.008), 0.656–0.687/0.657–0.688	0.556/0.559 (±0.016/0.016), 0.525–0.586/0.527–0.592	0.564/0.557 (±0.013/0.013), 0.539–0.590/0.530–0.583
**Long-term effect** (1st/last ECT(±SE), 95% CI)	0.636/0.634 (±0.007/0.009), 0.622–0.649/0.616–0.652	0.672/0.672 (±0.007/0.009), 0.658–0.685/0.655–0.690	0.572/0.543 (±0.014/0.019), 0.545–0.599/0.506–0.580	0.574/0.547 (±0.011/0.015), 0.552–0.595/0.517–0.577
**B cells (n=21)**				
**Acute effect** (before/after ECT(±SE), 95% CI)	0.634/0.634 (±0.008/0.008), 0.617–0.650/0.618–0.651	0.678/0.678 (±0.008/0.008), 0.662–0.695/0.661–0.694	0.535/0.540 (±0.017/0.017), 0.501–0.569/0.506–0.574	0.527/0.532 (±0.014/0.013), 0.500–0.554/0.505–0.558
**Long-term effect** (1st/last ECT(±SE), 95% CI)	0.632/0.636 (±0.008/0.009), 0.617–0.647/0.618–0.654	0.680/0.676 (±0.008/0.009), 0.665–0.695/0.658–0.694	0.529/0.546 (±0.016/0.019), 0.498–0.560/0.508–0.583	0.513/0.545 (±0.012/0.015), 0.489–0.537/0.516–0.575
**Monocytes (n=21)**				
**Acute effect** (before/after ECT(±SE), 95% CI)	0.389/0.387 (±0.009/0.009), 0.371–0.407/0.370–0.405	0.450/0.449 (±0.009/0.009), 0.433–0.468/0.432–0.467	0.089/0.073 (±0.011/0.011), 0.068–0.110/0.052–0.094	0.140/0.119 (±0.013/0.013), 0.116–0.165/0.094–0.144
**Long-term effect** (1st/last ECT(±SE), 95% CI)	0.388/0.389 (±0.008/0.010), 0.372–0.404/0.369–0.408	0.450/0.449 (±0.008/0.010), 0.435–0.466/0.430–0.468	0.064/0.098 (±0.010/0.011), 0.045–0.084/0.075–0.121*	0.109/0.150 (±0.012/0.014), 0.086–0.132/0.124–0.177*
**NK cells (n=21)**				
**Acute effect** (before/after ECT(±SE), 95% CI)	0.606/0.601 (±0.009/0.009), 0.589–0.623/0.584–0.618	0.641/0.634 (±0.009/0.009), 0.623–0.659/0.616–0.652	0.562/0.569 (±0.013/0.014), 0.535–0.589/0.543–0.596	0.548/0.552 (±0.011/0.011), 0.526–0.570/0.530–0.575

**Long-term effect** (1st/last ECT(±SE), 95% CI)	0.601/0.606 (±0.008/0.009), 0.585–0.616/0.588–0.625	0.636/0.639 (±0.008/0.010), 0.620–0.653/0.619–0.659	0.558/0.574 (±0.012/0.015), 0.533–0.582/0.545–0.603	0.540/0.559 (±0.010/0.012), 0.520–0.561/0.535–0.584
**T cells (n=21)**				
**Acute effect** (before/after ECT(±SE), 95% CI)	0.775/0.771 (±0.007/0.007), 0.762–0.789/0.758–0.785	0.793/0.791 (±0.007/0.007), 0.780–0.807/0.778–0.805	0.852/0.830 (±0.008/0.008), 0.837–0.867/0.815–0.846	0.825/0.799 (±0.008/0.008), 0.810–0.841/0.784–0.815*
**Long-term effect** (1st/last ECT(±SE), 95% CI)	0.772/0.774 (±0.006/0.008), 0.760–0.784/0.759–0.789	0.791/0.794 (±0.006/0.007), 0.779–0.803/0.779–0.808	0.848/0.835 (±0.007/0.008), 0.834–0.861/0.818–0.851	0.818/0.807 (±0.007/0.009), 0.804–0.832/0.790–0.823
**ECT Remitters**	**Total t-PA**	**MHRE**	**CREB**	**CREB + CTF/NF1**
**Blood (n=28)**				
**Acute effect** (before/after ECT(±SE), 95% CI)	0.474/0.484 (±0.006/0.006), 0.461–0.486/0.471–0.496	0.514/0.522 (±0.007/0.007), 0.501–0.527/0.509–0.535	0.352/0.398 (±0.012/0.012), 0.329–0.375/0.375–0.421	0.366/0.401 (±0.010/0.010), 0.347–0.385/0.382–0.420
**Long-term effect** (1st/last main. ECT(±SE), 95% CI)	0.483/0.465 (±0.009/0.010), 0.465–0.501/0.446–0.484^^	0.519/0.506 (±0.009/0.010), 0.501–0.537/0.486–0.526^^	0.397/0.344 (±0.017/0.018), 0.364–0.429/0.308–0.380^^	0.409/0.354 (±0.014/0.015), 0.383–0.436/0.325–0.383^^
**PBMCs (n=21)**				
**Acute effect** (before/after ECT(±SE), 95% CI)	0.651/0.644 (±0.011/0.011), 0.629–0.672/0.623–0.665	0.692/0.688 (±0.011/0.011), 0.671–0.713/0.667–0.709	0.556/0.558 (±0.022/0.022), 0.514–0.599/0.516–0.601	0.560/0.547 (±0.017/0.017), 0.525–0.594/0.512–0.581
**Long-term effect** (1st/last ECT(±SE), 95% CI)	0.648/0.647 (±0.010/0.011), 0.628–0.668/0.624–0.669	0.687/0.693 (±0.010/0.011), 0.668–0.707/0.670–0.715	0.579/0.536 (±0.020/0.023), 0.539–0.619/0.490–0.581	0.571/0.536 (±0.016/0.019), 0.538–0.603/0.499–0.572
**B cells (n=21)**				
**Acute effect** (before/after ECT(±SE), 95% CI)	0.627/0.636 (±0.012/0.012), 0.604–0.650/0.613–0.659	0.675/0.682 (±0.012/0.012), 0.652–0.699/0.659–0.705	0.511/0.546 (±0.024/0.024), 0.463–0.559/0.498–0.594	0.493/0.524 (±0.019/0.019), 0.456–0.530/0.488–0.561
**Long-term effect** (1st/last ECT(±SE), 95% CI)	0.628/0.635 (±0.011/0.012), 0.606–0.651/0.611–0.659	0.678/0.680 (±0.011/0.012), 0.655–0.700/0.656–0.703	0.529/0.528 (±0.024/0.025), 0.483–0.576/0.478–0.577	0.505/0.513 (±0.018/0.019), 0.469–0.540/0.475–0.551
**Monocytes (n=21)**				
**Acute effect** (before/after ECT(±SE), 95% CI)	0.398/0.403 (±0.013/0.013), 0.373–0.423/0.378–0.428	0.461/0.467 (±0.013/0.013), 0.436–0.485/0.442–0.491	0.085/0.081 (±0.015/0.015), 0.055–0.115/0.051–0.111	0.132/0.132 (±0.018/0.018), 0.097–0.167/0.097–0.167
**Long-term effect** (1st/last ECT(±SE), 95% CI)	0.395/0.406 (±0.012/0.013), 0.371–0.420/0.380–0.431	0.458/0.469 (±0.012/0.013), 0.434–0.482/0.444–0.495	0.064/0.098 (±0.010/0.011), 0.032–0.093/0.074–0.134*	0.110/0.154 (±0.018/0.018), 0.074–0.145/0.119–0.189
**NK cells (n=21)**				
**Acute effect** (before/after ECT(±SE), 95% CI)	0.622/0.608 (±0.012/0.012), 0.599–0.646/0.584–0.632	0.662/0.647 (±0.013/0.013), 0.637–0.687/0.622–0.672	0.567/0.551 (±0.019/0.019), 0.529–0.604/0.513–0.590	0.542/0.527 (±0.016/0.016), 0.511–0.572/0.496–0.559

**Long-term effect**<br>(1st/last ECT(±SE), 95% CI)	0.612/0.619 (±0.012/0.012),<br>0.589–0.635/0.594–0.643	0.650/0.659 (±0.012/0.013),<br>0.626–0.675/0.634–0.685	0.559/0.559 (±0.019/0.019),<br>0.522–0.596/0.521–0.597	0.533/0.536 (±0.016/0.016),<br>0.502–0.563/0.505–0.568
**T cells (n=21)**				
**Acute effect**<br>(before/after ECT(±SE), 95% CI)	0.782/0.778 (±0.010/0.010),<br>0.763–0.801/0.759–0.797	0.805/0.802 (±0.010/0.010),<br>0.786–0.823/0.784–0.821	0.844/0.827 (±0.011/0.011),<br>0.823–0.865/0.805–0.849	0.813/0.789 (±0.011/0.011),<br>0.792–0.835/0.766–0.811
**Long-term effect**<br>(1st/last ECT(±SE), 95% CI)	0.780/0.781 (±0.009/0.010),<br>0.761–0.798/0.761–0.800	0.804/.0.803 (±0.009/0.010),<br>0.786–0.823/0.783–0.822	0.836/0.834 (±0.011/0.011),<br>0.815–0.857/0.812–0.856	0.800/0.802 (±0.011/0.011),<br>0.778–0.821/0.780–0.825
**ECT non-remitters**	**Total t-PA**	**MHRE**	**CREB**	**CREB + CTF/NF1**
**Blood (n=28)**				
**Acute effect**<br>(before/after ECT(±SE), 95% CI)	0.459/0.459 (±0.009/0.009),<br>0.442–0.476/0.442–0.476	0.491/0.489 (±0.009/0.009),<br>0.473–0.509/0.471–0.507	0.392/0.395 (±0.016/0.016),<br>0.360–0.424/0.363–0.427	0.403/0.407 (±0.013/0.013),<br>0.376–0.429/0.381–0.433
**Long-term effect**<br>(1st/last main. ECT(±SE), 95% CI)	0.458/0.475 (±0.008/0.018),<br>0.441–0.474/0.440–0.510[^^]	0.488/0.507 (±0.009/0.018),<br>0.471–0.504/0.470–0.543[^^]	0.400/0.396 (±0.015/0.033),<br>0.371–0.430/0.332–0.461[^^]	0.408/0.419 (±0.013/0.027),<br>0.384–0.433/0.367–0.472[^^]
**PBMCs (n=21)**				
**Acute effect**<br>(before/after ECT(±SE), 95% CI)	0.619/0.626 (±0.012/0.012),<br>0.595–0.643/0.602–0.650	0.650/0.657 (±0.012/0.012),<br>0.627–0.674/0.633–0.681	0.555/0.560 (±0.024/0.026),<br>0.507–0.602/0.509–0.612	0.569/0.567 (±0.020/0.021),<br>0.529–0.608/0.525–0.608
**Long-term effect**<br>(1st/last ECT(±SE), 95% CI)	0.624/0.621 (±0.010/0.014),<br>0.603–0.644/0.593–0.649	0.656/0.651 (±0.010/0.014),<br>0.636–0.677/0.624–0.679	0.565/0.550 (±0.021/0.030),<br>0.524–0.607/0.491–0.609	0.577/0.559 (±0.017/0.025),<br>0.543–0.610/0.510–0.607
**B cells (n=21)**				
**Acute effect**<br>(before/after ECT(±SE), 95% CI)	0.640/0.632 (±0.013/0.013),<br>0.615–0.665/0.607–0.657	0.681/0.674 (±0.013/0.013),<br>0.656–0.707/0.648–0.699	0.558/0.534 (±0.027/0.027),<br>0.506–0.611/0.482–0.587	0.561/0.539 (±0.021/0.021),<br>0.520–0.602/0.498–0.580
**Long-term effect**<br>(1st/last ECT(±SE), 95% CI)	0.636/0.636 (±0.011/0.014),<br>0.613–0.658/0.608–0.664	0.682/0.673 (±0.012/0.014),<br>0.659–0.704/0.645–0.702	0.529/0.564 (±0.024/0.030),<br>0.482–0.575/0.506–0.623	0.522/0.578 (±0.018/0.024),<br>0.486–0.558/0.531–0.624
**Monocytes (n=21)**				
**Acute effect**<br>(before/after ECT(±SE), 95% CI)	0.380/0.372 (±0.014/0.014),<br>0.353–0.408/0.344–0.399	0.440/0.431 (±0.014/0.014),<br>0.413–0.467/0.405–0.458	0.093/0.065 (±0.016/0.016),<br>0.061–0.125/0.033–0.097	0.149/0.106 (±0.019/0.020),<br>0.111–0.187/0.067–0.146
**Long-term effect**<br>(1st/last ECT(±SE), 95% CI)	0.380/0.372 (±0.012/0.016),<br>0.356–0.405/0.341–0.402	0.443/0.428 (±0.012/0.015),<br>0.419–0.467/0.398–0.458	0.066/0.092 (±0.015/0.018),<br>0.037–0.095/0.056–0.127	0.109/0.147 (±0.018/0.022),<br>0.074–0.144/0.104–0.189
**NK cells (n=21)**				
**Acute effect**<br>(before/after ECT(±SE), 95% CI)	0.589/0.594 (±0.013/0.013),<br>0.563–0.615/0.568–0.620	0.620/0.621 (±0.014/0.014),<br>0.592–0.647/0.594–0.649	0.557/0.588 (±0.021/0.021),<br>0.516–0.598/0.547–0.629	0.554/0.577 (±0.017/0.017),<br>0.520–0.587/0.542–0.611

**Long-term effect** (1st/last ECT(±SE), 95% CI)	0.589/0.594 (±0.012/0.015), 0.566–0.613/0.565–0.623	0.623/0.619 (±0.012/0.016), 0.598–0.647/0.588–0.649	0.557/0.589 (±0.018/0.023), 0.520–0.593/0.543–0.634	0.548/0.583 (±0.015/0.019), 0.518–0.578/0.545–0.621
**T cells (n=21)**				
**Acute effect** (before/after ECT(±SE), 95% CI)	0.768/0.764 (±0.011/0.011), 0.747–0.789/0.743–0.785	0.782/0.780 (±0.010/0.010), 0.761–0.802/0.760–0.801	0.860/0.834 (±0.012/0.012), 0.837–0.883/0.811–0.857	0.838/0.810 (±0.012/0.012), 0.814–0.861/0.786–0.833
**Long-term effect** (1st/last ECT(±SE), 95% CI)	0.765/0.767 (±0.009/0.012), 0.746–0.783/0.744–0.790	0.777/0.785 (±0.009/0.012), 0.759–0.795/0.762–0.808	0.859/0.835 (±0.010/0.013), 0.838–0.879/0.809–0.861	0.836/0.811 (±0.011/0.013), 0.815–0.857/0.785–0.837

Blood

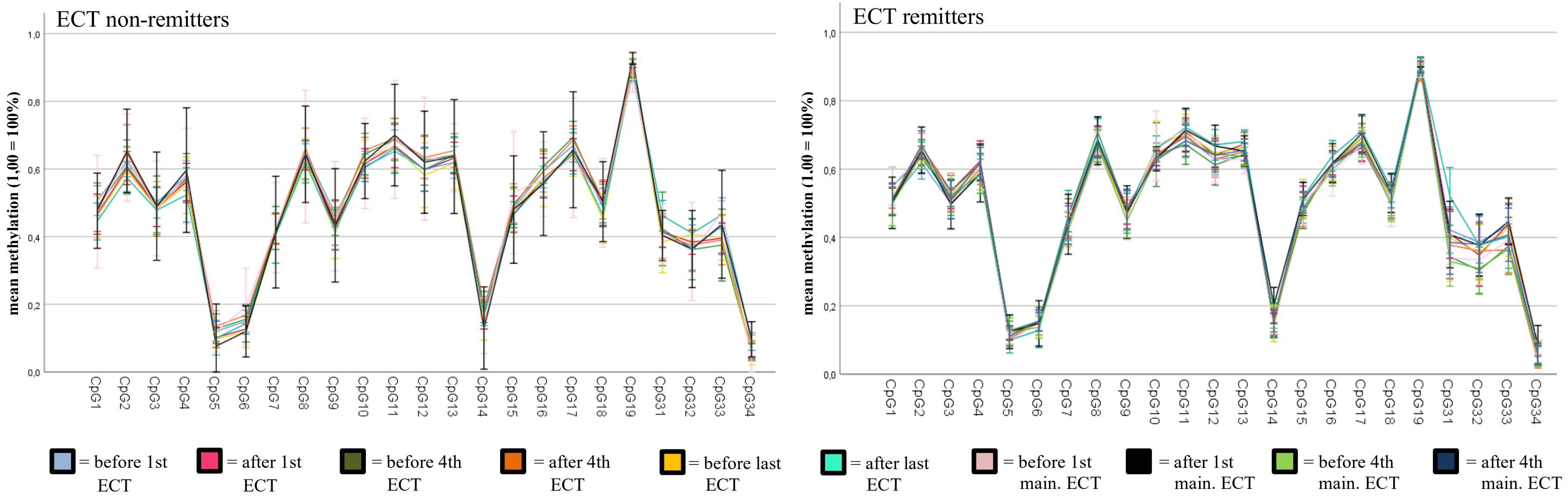
ECT non-remitters
ECT remitters
mean methylation (1,00 = 100%)
0,0
0,2
0,4
0,6
0,8
1,0
CpG1 CpG2 CpG3 CpG4 CpG5 CpG6 CpG7 CpG8 CpG9 CpG10 CpG11 CpG12 CpG13 CpG14 CpG15 CpG16 CpG17 CpG18 CpG19 CpG31 CpG32 CpG33 CpG34
= before 1st ECT
= after 1st ECT
= before 4th ECT
= after 4th ECT
= before last ECT
= after last ECT
= before 1st main. ECT
= after 1st main. ECT
= before 4th main. ECT
= after 4th main. ECT

PBMCs

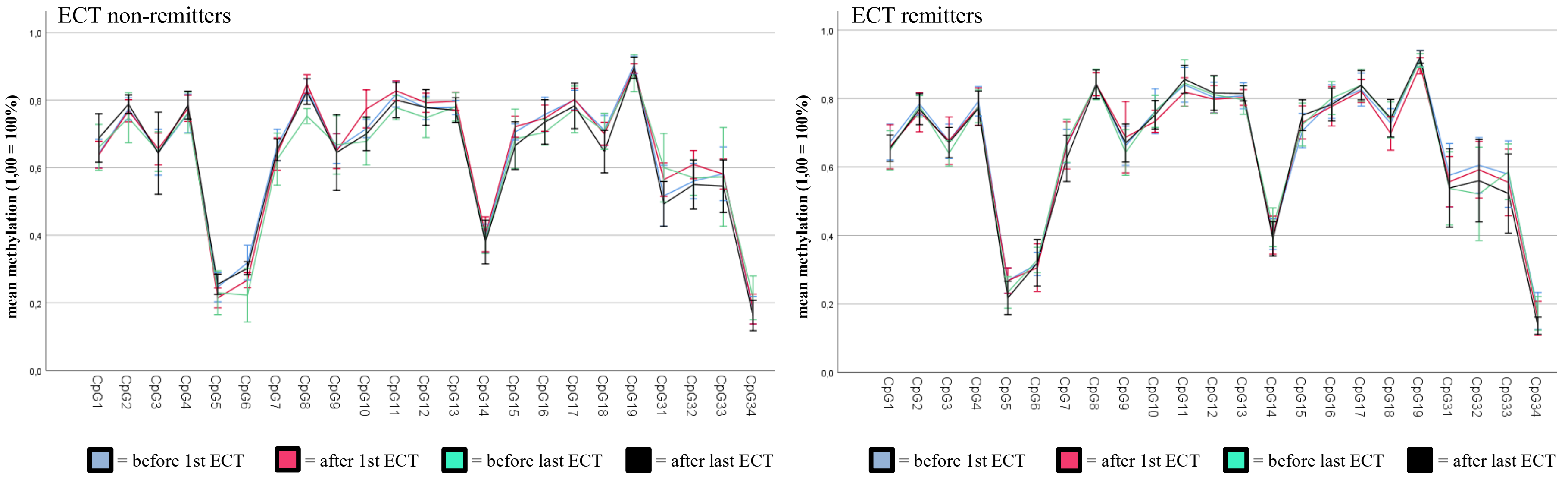
ECT non-remitters
ECT remitters
mean methylation (1,00 = 100%)
0,0
0,2
0,4
0,6
0,8
1,0
CpG1 CpG2 CpG3 CpG4 CpG5 CpG6 CpG7 CpG8 CpG9 CpG10 CpG11 CpG12 CpG13 CpG14 CpG15 CpG16 CpG17 CpG18 CpG19 CpG31 CpG32 CpG33 CpG34
= before 1st ECT
= after 1st ECT
= before last ECT
= after last ECT

B cells

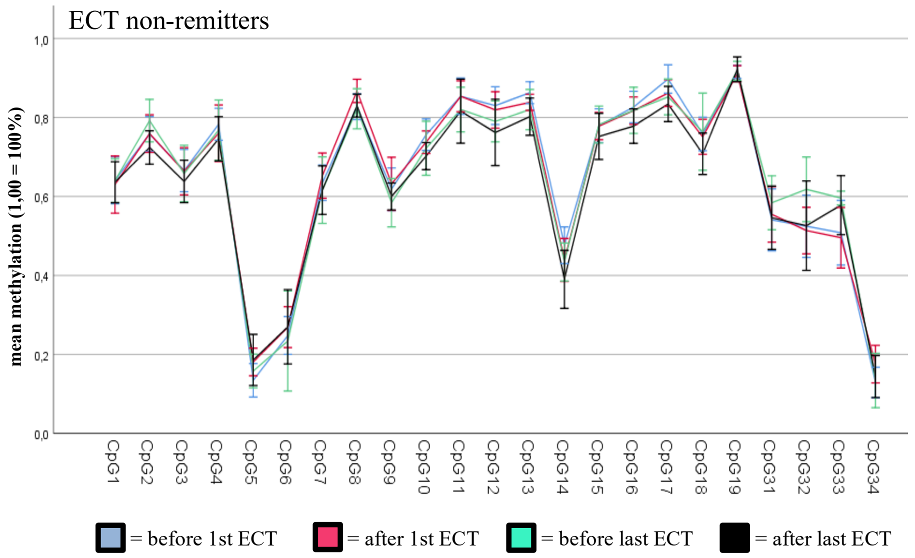

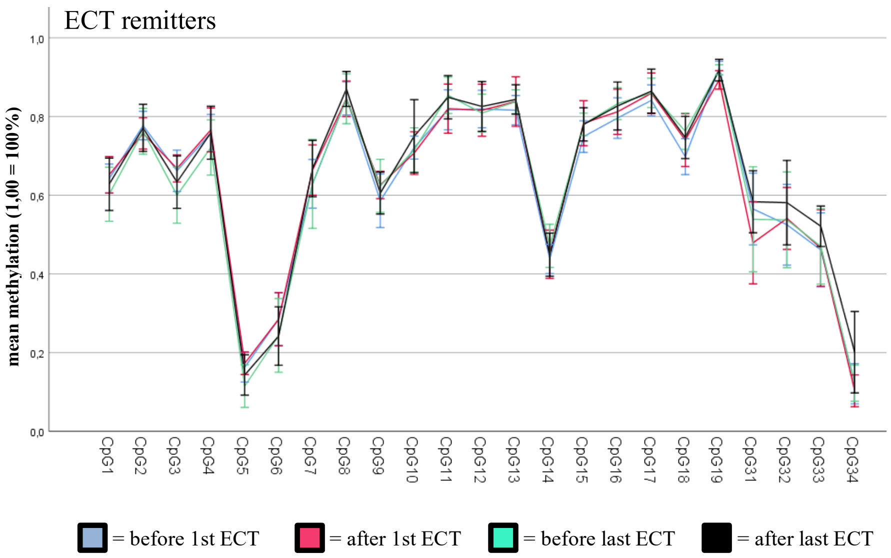

Monocytes

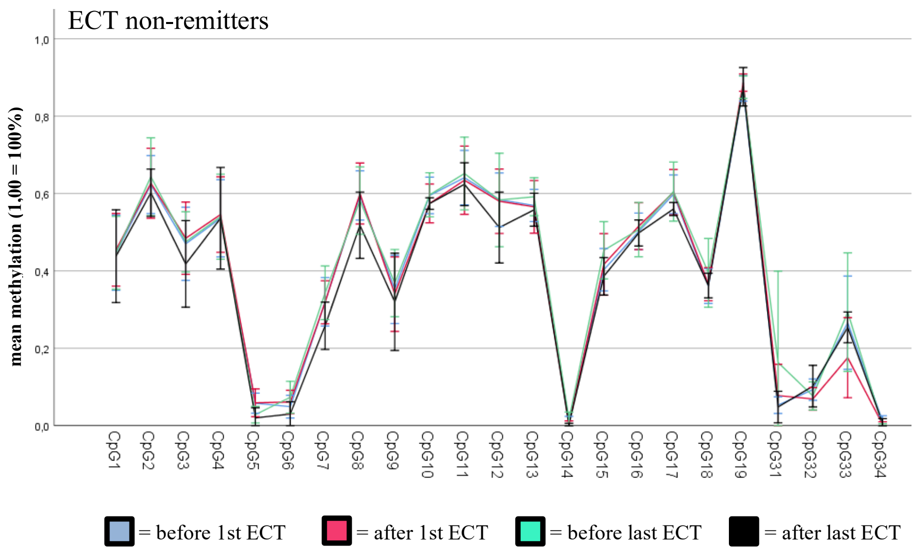

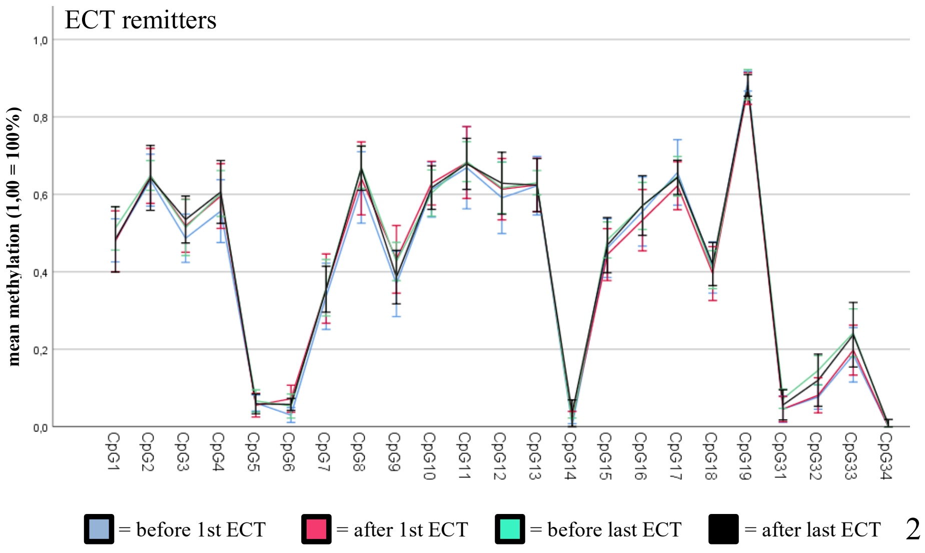

NK cells

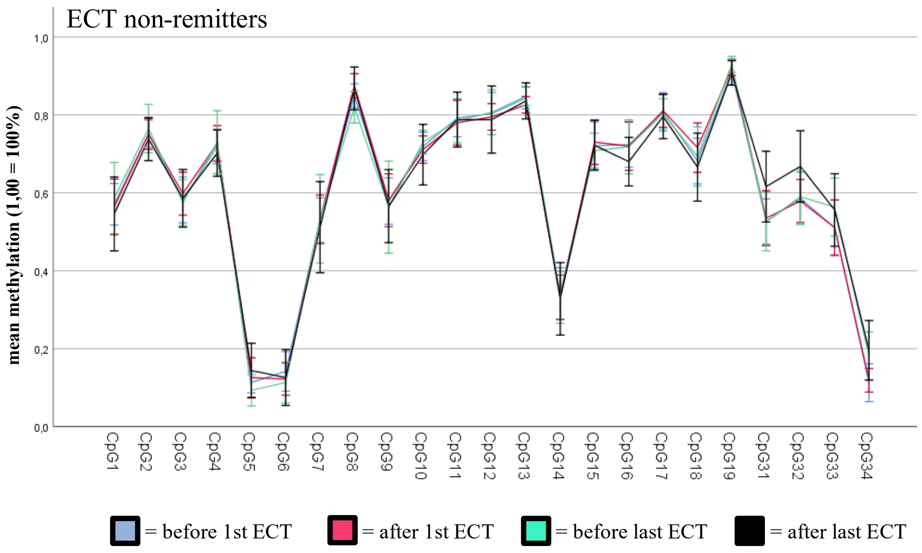

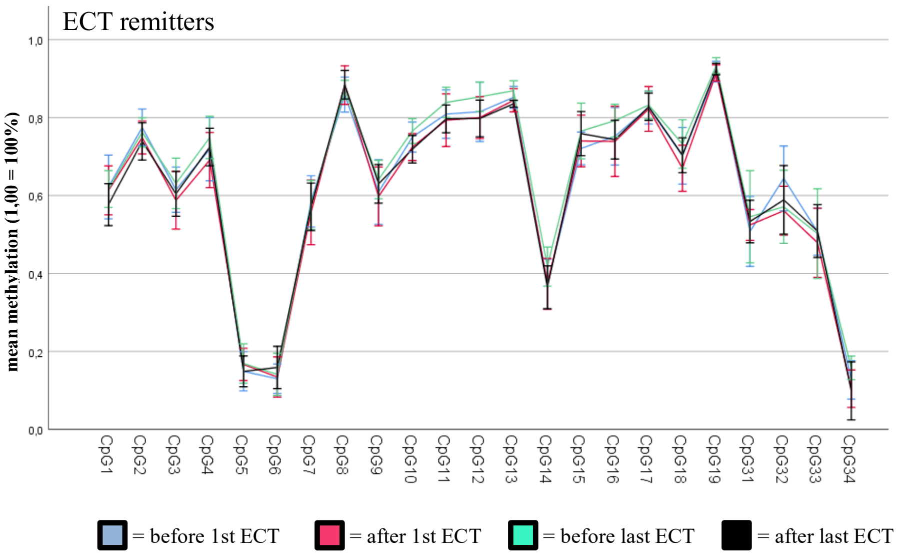

T cells

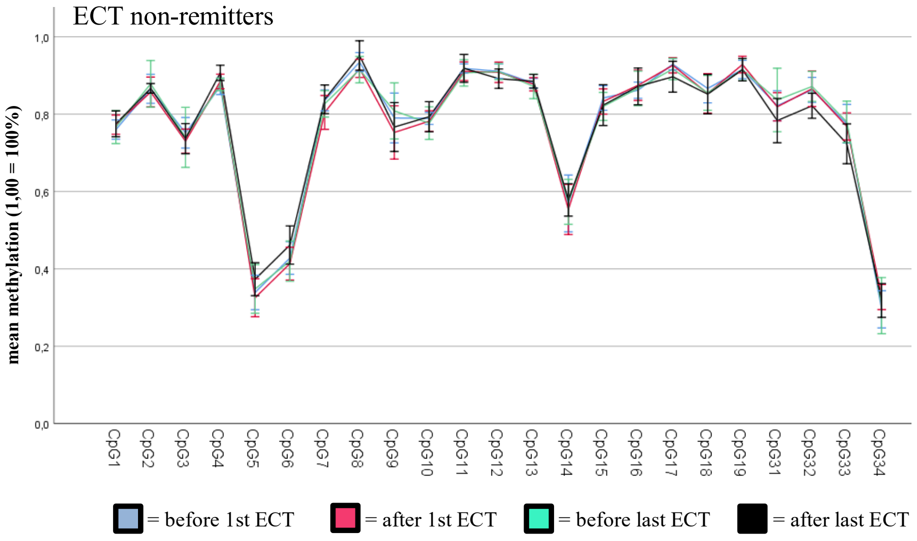

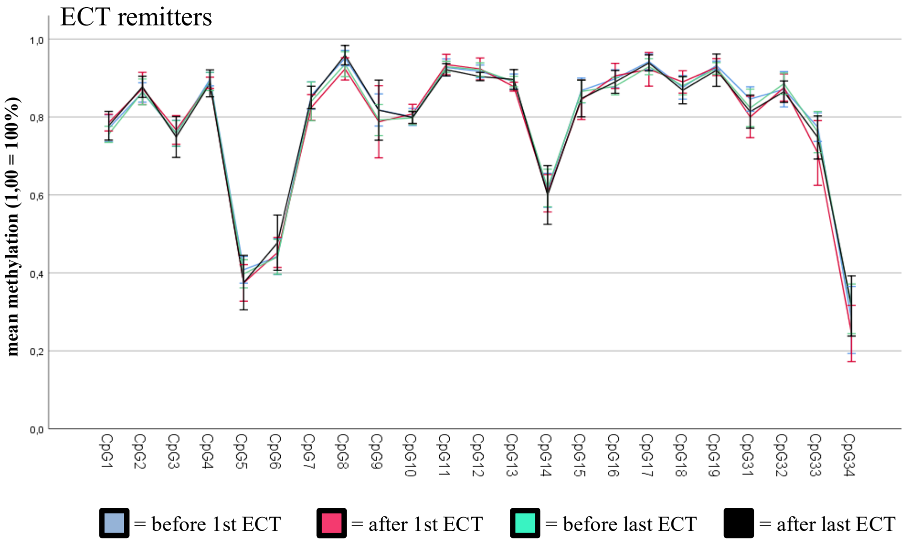

Figure S4: ***DNA methylation changes over the time course of ECT.*** The DNA methylation of defined t-PA gene regions changed upon a course of ECT (see Tables S10 and S11). Results were calculated via mixed linear models and corrected with Sidak's post-hoc test. They are presented as mean (± double standard error).
